# An Autonomous Oscillation Times and Executes Centriole Biogenesis

**DOI:** 10.1016/j.cell.2020.05.018

**Published:** 2020-06-25

**Authors:** Mustafa G. Aydogan, Thomas L. Steinacker, Mohammad Mofatteh, Zachary M. Wilmott, Felix Y. Zhou, Lisa Gartenmann, Alan Wainman, Saroj Saurya, Zsofia A. Novak, Siu-Shing Wong, Alain Goriely, Michael A. Boemo, Jordan W. Raff

**Affiliations:** 1 Sir William Dunn School of Pathology, University of Oxford, Oxford OX1 3RE, UK; 2 Mathematical Institute, University of Oxford, Oxford OX2 6GG, UK; 3 Ludwig Institute for Cancer Research, University of Oxford, Oxford OX3 7DQ, UK

**Keywords:** centriole, centriole duplication, centrosome, cell cycle, organelle biogenesis, organelle sizing, biological oscillator, biological timing, FCS

## Abstract

The accurate timing and execution of organelle biogenesis is crucial for cell physiology. Centriole biogenesis is regulated by Polo-like kinase 4 (Plk4) and initiates in S-phase when a daughter centriole grows from the side of a pre-existing mother. Here, we show that a Plk4 oscillation at the base of the growing centriole initiates and times centriole biogenesis to ensure that centrioles grow at the right time and to the right size. The Plk4 oscillation is normally entrained to the cell-cycle oscillator but can run autonomously of it—potentially explaining why centrioles can duplicate independently of cell-cycle progression. Mathematical modeling indicates that the Plk4 oscillation can be generated by a time-delayed negative feedback loop in which Plk4 inactivates the interaction with its centriolar receptor through multiple rounds of phosphorylation. We hypothesize that similar organelle-specific oscillations could regulate the timing and execution of organelle biogenesis more generally.

## Highlights

• Centriolar Plk4 levels oscillate and act as a switch for centriole biogenesis
• Oscillations may be generated via an Asl/Plk4 delayed negative feedback loop
• Plk4 oscillations are entrained and phase-locked by the Cdk/Cyclin oscillator (CCO)
• Plk4 oscillations can drive centriole biogenesis even when the CCO is perturbed

## Graphical Abstract

**Figure:**
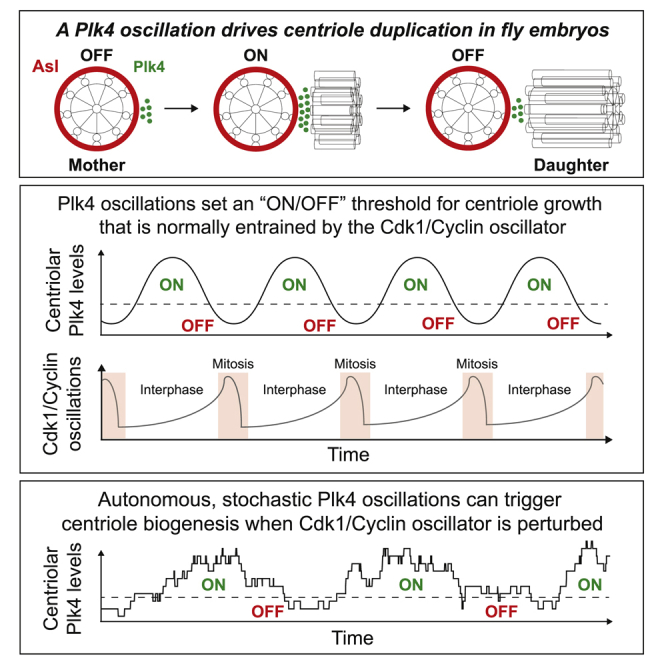

## Teaser

Feedback-driven oscillations in centriolar Plk4 kinase levels—normally entrained by the cell-cycle oscillator but capable of running autonomously—trigger and time centriole biogenesis to ensure that daughter centrioles grow at the right time and to the right size.

## Introduction

Albert Claude’s landmark paper (Claude, 1943) challenged the idea that cells are a mere bag of enzymes whose contents grow freely in the cytoplasm with no active regulation. We now appreciate the diverse and compact nature of the many organelles in the cytoplasm (Marsh et al., 2001), yet the physical mechanisms that regulate the number and size of these organelles remain largely unknown (Marshall, 2016). For most organelles in the cell, however, this question has been difficult to address, as the variation in their numbers and 3D-shape has made it challenging to monitor their growth—or to even determine which parameter (e.g., their surface area, volume, or perhaps the amount of a limiting component) best defines their size.

Centrioles are highly structured organelles that form centrosomes and cilia (Bettencourt-Dias et al., 2011, Nigg and Holland, 2018, Nigg and Raff, 2009). Their linear structure and tightly controlled pattern of duplication makes them an attractive model with which to study organelle biogenesis (Goehring and Hyman, 2012, Marshall, 2016). Most cells are born with a single pair of centrioles that duplicate precisely once during S-phase, when a daughter centriole grows out orthogonally from the base of each mother until it reaches the same size as its mother (Banterle and Gönczy, 2017, Fırat-Karalar and Stearns, 2014, Nigg and Holland, 2018). To monitor the dynamics of centriole growth, we recently examined living syncytial *Drosophila* embryos where we could follow the assembly of hundreds of centrioles as they duplicate in near-synchrony in a common cytoplasm (Aydogan et al., 2018). These studies revealed that centriole growth in these embryos is homeostatic: when centrioles grow slowly, they grow for a longer period; when centrioles grow quickly, they grow for a shorter period. As a result, centrioles grow to a consistent size.

Polo-like kinase 4 (Plk4) is the master regulator of centriole biogenesis and it is initially recruited to a ring around the mother centriole, but this ring resolves into a single focus on the side of the mother, defining the site of daughter centriole assembly (Banterle and Gönczy, 2017, Fırat-Karalar and Stearns, 2014, Leda et al., 2018, Nigg and Holland, 2018, Takao et al., 2019). Unexpectedly, we found that Plk4 not only determines the position of this site, but also helps to establish the inverse relationship between the rate and period of daughter centriole growth (Aydogan et al., 2018). Plk4 presumably influences the rate of centriole growth, at least in part, by phosphorylating Ana2/STIL to promote its interaction with Sas-6 and, consequently, the assembly of the central cartwheel (Dzhindzhev et al., 2014, Kratz et al., 2015, Ohta et al., 2014), the 9-fold symmetric structure that forms the backbone of the growing daughter centriole (Kitagawa et al., 2011, van Breugel et al., 2011, van Breugel et al., 2014). It is less clear, however, how Plk4 might influence the period of centriole growth.

Recent studies have shown that Plk4 localizes to centrioles in a cyclical manner in both fly embryos (Aydogan et al., 2018) and human cultured cells (Takao et al., 2019), but the functional significance of this localization pattern is unclear. Here, we show that a Plk4 oscillation at the base of the growing centriole initiates and times centriole biogenesis in fly embryos.

## Results and Discussion

### Plk4 Levels Oscillate at the Base of Growing Daughter Centrioles

**Figure 1:**
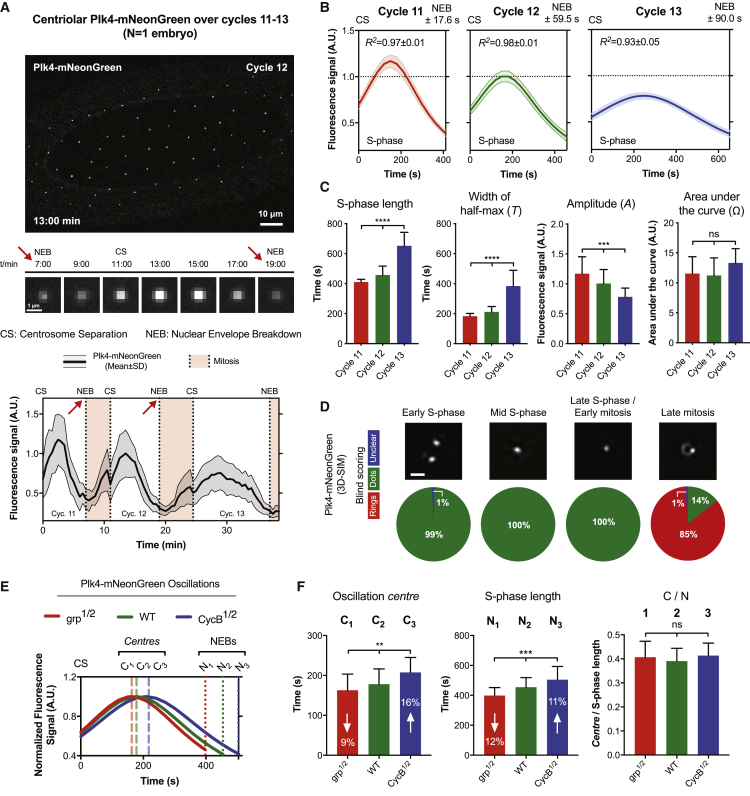
Plk4 Levels Oscillate at the Centriole in a Process Entrained by the CCO (A) Top panel: micrograph shows an image from a time-lapse movie of an embryo expressing Plk4-NG. Middle panels: micrographs illustrate the centriolar Plk4-NG oscillation during nuclear cycle 12—obtained by superimposing all the Plk4-NG foci (n = 60) at each time point (see STAR Methods). Bottom panel: quantification of centriolar Plk4-NG levels during nuclear cycles 11–13 in a single embryo (red arrows highlight equivalent time points in the middle panels). (B) Graphs show the mathematical regression of centriolar Plk4-NG dynamics during S-phase of cycles 11–13 (regression mean ± SEM). R^2^ values indicate goodness-of-fit. N ≥ 15 embryos; n = 24, 37, and 53 centrioles (mean) per embryo over cycles 11–13, respectively. (C) The bar charts quantify the oscillation parameters—derived from the data shown in (B). Data are presented as mean ± SD. Statistical significance was assessed using an ordinary one-way ANOVA test (for Gaussian-distributed data) or a Kruskal-Wallis test (^∗∗∗^p < 0.001; ^∗∗∗∗^p < 0.0001; ns, not significant). (D) Micrographs show, and pie charts quantify, the distribution of Plk4-NG at centrioles assessed by 3D-SIM at the indicated phases of the nuclear cycle (see STAR Methods). N = 6 embryos per cell-cycle stage; n = 20 centrioles per embryo; all images were scored blindly by 3 assessors and the mean score is shown (scale bar, 0.5 μm). (E) Graph shows the mean regression of Plk4-NG oscillations in nuclear cycle 12 of WT embryos (green), or in embryos where the genetic dose of either cyclin B (*CycB*^*1/2*^; blue) or grapes (*Drosophila* Chk1) (*grp*^*1/2*^; red) has been halved to slow or speed-up the nuclear cycles, respectively. Dashed lines mark the center (peak) of the Plk4-NG oscillations (denoted with C), and dotted lines indicate the time of NEB (denoted with N) for each genotype. N ≥ 14 embryos for each condition; n = 55, 43, and 44 centrioles (mean) per embryo in WT, *CycB*^*1/2*^, and *grp*^*1/2*^ embryos, respectively. To clearly illustrate the phase shift in the oscillations, the highest mean fluorescence signal for each group was normalized to 1. (F) Bar charts quantify the time at which the Plk4-NG oscillations peaked, the length of S-phase, and the ratio between them (C/N)—derived from the data shown in (E). Data are presented as mean ± SD. Statistical significance was assessed using an ordinary one-way ANOVA test (for Gaussian-distributed data) or a Kruskal-Wallis test (^∗∗^p < 0.01; ^∗∗∗^p < 0.001; ns, not significant). See also Figures 6, S1, and S2.

**Figure S1:**
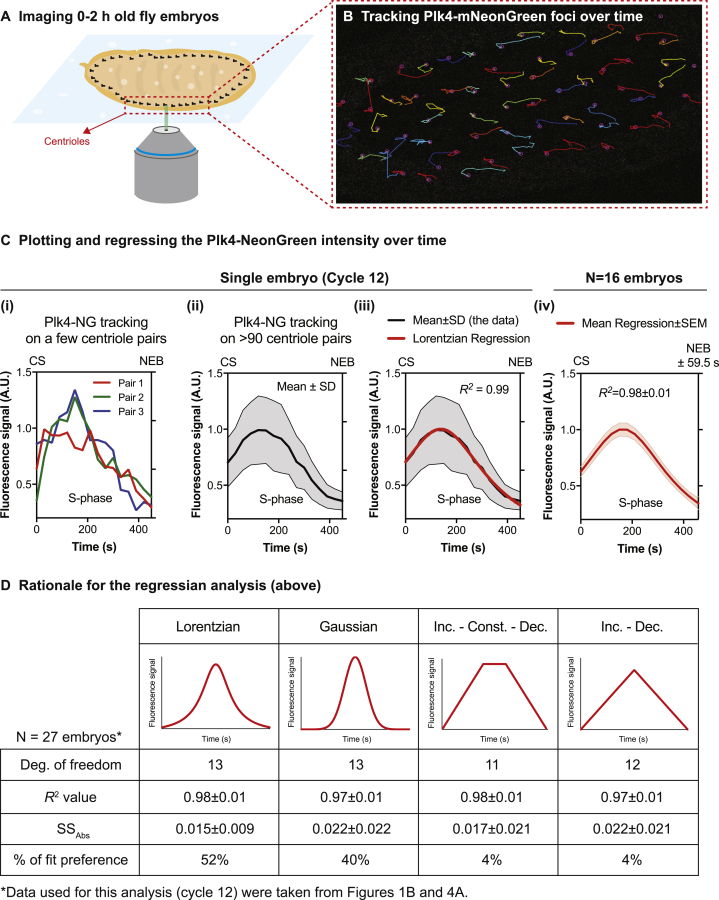
Summary of the Protocol for Image Acquisition, Processing, and Analysis of the Plk4-NG Oscillations, Related to Figure 1 (A) Diagram illustrates the centrioles in ~2 h old embryo expressing Plk4-NG being imaged on a spinning-disk confocal system. (B) Micrograph shows a typical image of the tracks of the Plk4-NG centrioles in S-phase of cycle 12, tracked using the ImageJ plugin, TrackMate. (C) Graphs show the Plk4-NG oscillation during cycle 12 in a single embryo quantified from the tracks of either several individual centriole pairs (i), or the Mean ± SD oscillation calculated from the tracks of > 90 centriole pairs (ii). The data for each embryo was then regressed using a Lorentzian equation (*red* line, iii)—see (D) for an explanation of the rationale for choosing this function. This process was repeated for multiple embryos to calculate a Mean ± SEM regression for nuclear cycle 12 (iv). *R*^*2*^ values indicate the goodness-of-fit (Mean ± SD) of the regression. CS = time of centrosome separation (set to 0); NEB = time of nuclear envelope breakdown. (D) Table shows the various models that were tested to fit the Plk4-NG oscillation data. *R*^*2*^ and *SS*_*Abs*_ (absolute sum of squares) values indicate the goodness of fit. The Lorentzian function was the best fit for the majority of embryos, so it was used for all further analyses. Further details of these models are provided in STAR Methods.

**Figure S2:**
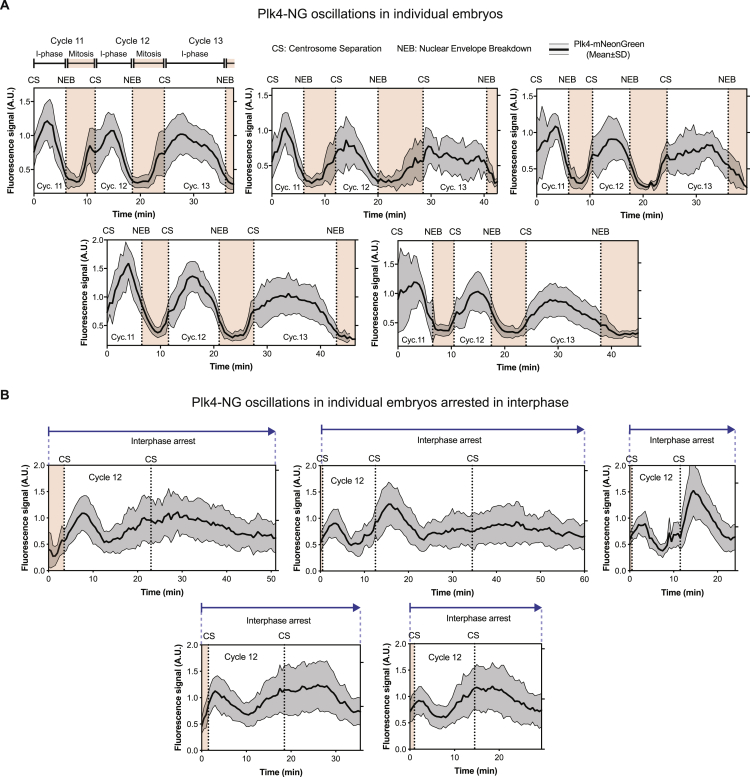
Plk4-NG Oscillations in Individual Embryos, Related to Figures 1 and 5 (A) Graphs show the Mean ± SD centriolar fluorescence intensity of Plk4-NG (two copies of a transgene expressed from its own promoter in a *Plk4* null mutant background) during nuclear cycles 11-13 in 5 different embryos imaged on a spinning-disk confocal system. n = 26 centrioles (mean) tracked starting from cycle 11 per embryo. See STAR Methods for full details of image acquisition and data analysis. (B) Same as in (A), but showing the Plk4-NG oscillation in 5 embryos arrested in interphase by the injection of dsRNAs against Cyclin A, B and B3. See Figure 5A for further details on sample numbers and experimental protocol.

To investigate the cyclical recruitment of Plk4 to the centrioles, we generated flies transgenically expressing Plk4-mNeonGreen (Plk4-NG) under the control of its own promoter in a *Plk4* mutant background. We monitored centriolar Plk4-NG levels in living *Drosophila* syncytial embryos, where the duration of S-phase gradually elongates over nuclear cycles 11–13 (Figures 1A, S1, and S2A; Video S1). Centriolar Plk4-NG levels oscillated during each cycle: levels started to rise in M-phase, peaked in early-mid S-phase, and were minimal by the next M-phase (Figures 1A and S2A). We fit the S-phase oscillations in individual embryos (Figures S1C and S1D) to derive an average S-phase oscillation for each cycle (Figure 1B).

Video S1. Monitoring Plk4-NG Oscillations in a *Drosophila* Embryo, Related to Figure 1 Time-lapse video of an embryo expressing two copies of Plk4-NG (expressed transgenically from the endogenous *Plk4* promoter) in a *Plk4* mutant background, observed on a spinning-disk confocal microscope through nuclear cycles 11-13. The movie is a maximum-intensity projection that has been photo-bleach corrected, but not background subtracted for visual clarity. Time (min:sec) is shown at the top left, and stage of the cell cycle is indicated at the bottom left.

Not surprisingly, the Plk4 oscillations appeared to be entrained by the core Cdk/Cyclin oscillator as their period increased as nuclear cycles slowed during cycles 11–13 (Figure 1C). Moreover, genetically altering the duration of the nuclear cycles elicited corresponding alterations in the Plk4 oscillation period (Figures 1E and 1F). Interestingly, however, the Plk4 oscillation exhibited adaptive behavior: as the period (*T*) of the oscillation tended to increase at successive cycles, its amplitude (*A*) tended to decrease, so that the total amount of Plk4 recruited to centrioles—i.e., the area under the S-phase oscillation curve (area under the curve [Ω])—remained relatively constant (Figure 1C).

Plk4 is initially recruited to a ring around the mother centriole that resolves into a single hub that defines the site of daughter centriole assembly (Banterle and Gönczy, 2017, Fırat-Karalar and Stearns, 2014, Nigg and Holland, 2018). To examine how this localization related to the Plk4 oscillations, we used 3D-structured illumination super-resolution microscopy (3D-SIM) to assess the centriolar localization of Plk4 during the nuclear cycles in living embryos. Plk4-NG was only very briefly detectable in a ring during late-mitosis; at all other stages it appeared largely as a single hub (Figure 1D). Thus, the recruitment and loss of Plk4 from the centriole wall is not responsible for the S-phase oscillation we observe in these embryos; instead, centriolar Plk4-NG levels oscillate at the base of the growing daughter centriole.

### Plk4 Oscillations Time and Execute Centriole Biogenesis

**Figure S3:**
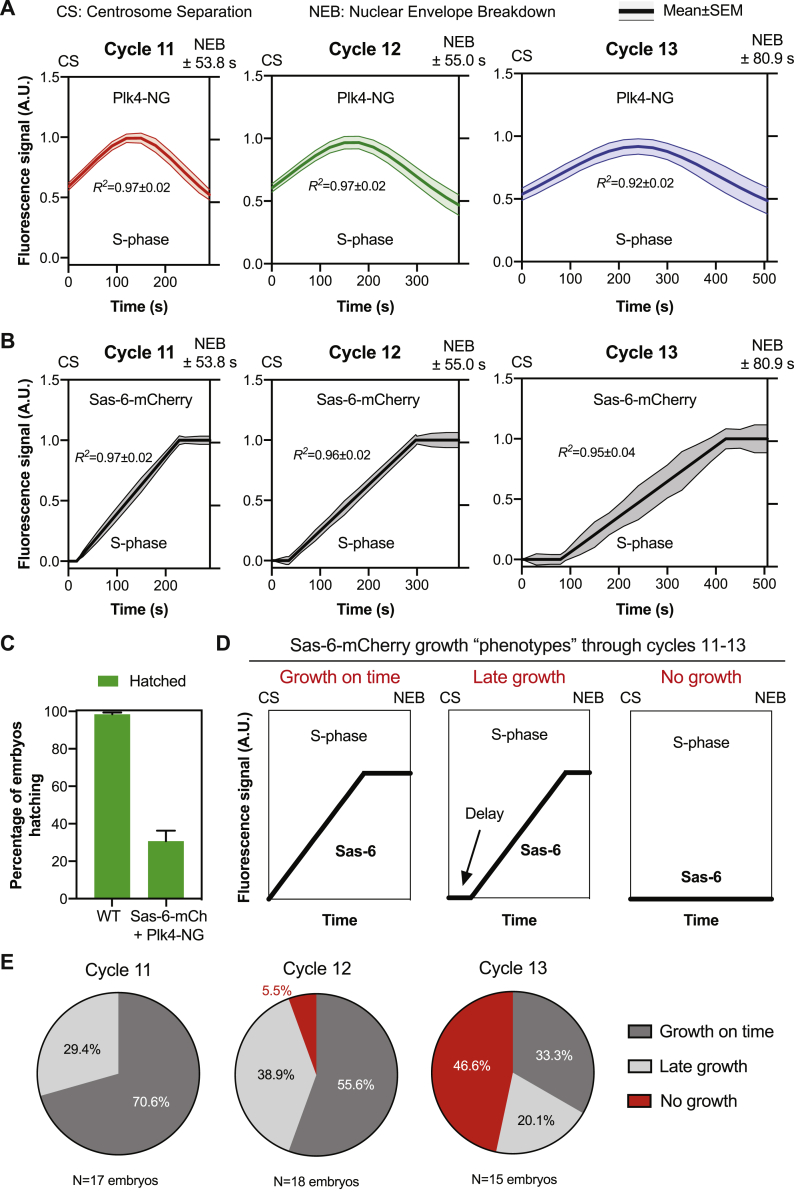
Simultaneously Measuring Centriole Growth and the Plk4 Oscillation in the Same Embryos, Related to Figure 2 (A and B) Graphs show the same data presented in Figure 2A, but with the SEM included (as these error bars were omitted from Figure 2A for ease of presentation). CS = centrosome separation and NEB = nuclear envelope breakdown. *R*^*2*^ values indicate the goodness of fit. (C) Graph quantifies the embryo hatching frequency in embryos laid by either wild-type (Oregon-R) females or females simultaneously expressing Sas-6-mCherry and Plk4-NG in a *Plk4* mutant background (all mated with WT males). At least 4 technical repeats were carried out over several days, and a total of at least 400 embryos were analyzed. (D) Cartoon graphs (i.e., imaginary data) illustrate the three different centriole growth phenotypes we observed in the *Plk4* mutant embryos that simultaneously express 2 copies of Plk4-NG and one copy of Sas-6-mCherry. In our previous analysis of centriole growth kinetics (Aydogan et al., 2018) almost all embryos started to incorporate Sas-6-GFP at the very start of S-phase (“Growth on time,” left graph). In the embryos analyzed here (with a more complicated genotype, and expressing Sas-6-mCherry rather than Sas-6-GFP), some of the embryos exhibited a clear delay in initiating the incorporation of Sas-6-mCherry (“Late growth,” middle graph), while others did not appear to incorporate significant amounts of Sas-6-mCherry at all (“No growth,” right graph). (E) Pie charts quantify the percentage of embryos exhibiting each centriole growth phenotype at each nuclear cycle. Note that embryos exhibiting the “No growth” phenotype were excluded from the analysis shown in (A) and (B) and in Figure 2A, although the amplitude of the Plk4 oscillations in these embryos was analyzed separately (Figure 2C): we observed 8 embryos in total that exhibited the “No growth” phenotype (1 in cycle 12, and 7 in cycle 13). Centriolar Plk4-NG levels continued to oscillate in these embryos, and the scatter graph shown in Figure 2C plots the peak amplitude of the Plk4-NG oscillations in these 8 embryos overlaid on the average “threshold” level of Plk4-NG at which centrioles started to grow in the population of embryos that did exhibit Sas-6-mCherry incorporation. This threshold was very similar at cycle 12 and 13, so the threshold shown in Figure 2C is taken from cycle 13 embryos (as 7 of the 8 embryos shown here were at cycle 13). The Plk4-NG oscillation in all but one of the 8 embryos failed to reach the average “threshold” level that would normally initiate centriole growth in these embryos.

**Figure 2:**
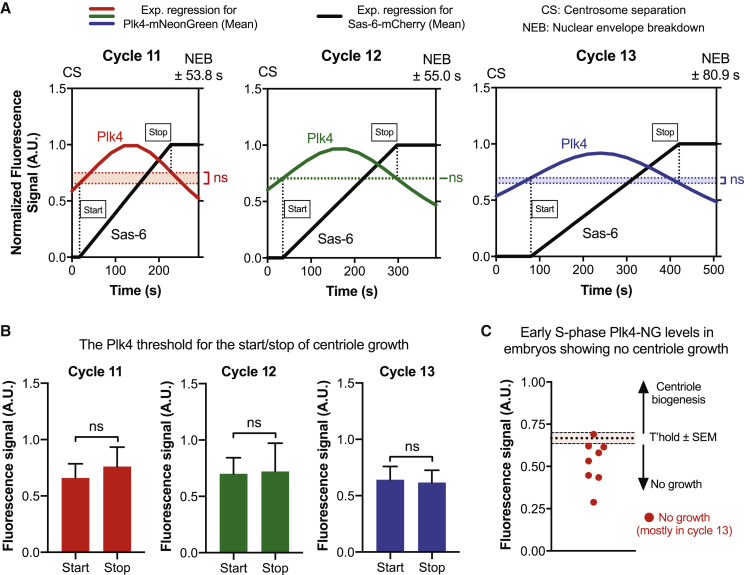
Plk4 Oscillations Initiate and Time Centriole Biogenesis (A) Graphs show the mean regression of Plk4-NG oscillations (red, green, and blue lines for cycles 11–13, respectively) and centriole growth (monitored by Sas-6-mCherry incorporation, black lines) measured simultaneously in embryos during S-phase of cycles 11–13. For ease of presentation, the SEM for these data are not shown, but are presented in Figures S3A and S3B. Dotted lines indicate the centriolar Plk4 levels at which centrioles “start” or “stop” growing. N = 17 embryos (cycles 11 and 12), and 8 embryos (cycle 13); n = 19, 31, and 45 centrioles (mean) per embryo in cycles 11–13, respectively. See STAR Methods for an explanation of data normalization and scaling. (B) Bar charts quantify the centriolar Plk4-NG threshold levels at which centrioles start and stop growing during cycles 11–13—derived from the data shown in (A). Data are presented as mean ± SD. Statistical significance was assessed using an unpaired t test with Welch’s correction (for Gaussian-distributed data) or an unpaired Mann-Whitney test (ns, not significant). (C) Eight embryos in which the centrioles did not grow (Figures S3D and S3E) were excluded from the analysis shown in (A) and (B); the scatter graph shown here illustrates how the mean amplitude of the Plk4 oscillations in each of these eight embryos (red dots) tended to be lower than the mean amplitude (±SEM) of the Plk4 oscillations in the embryos where the centrioles did grow. See also Figure S3.

To test whether the Plk4 oscillations were important for centriole biogenesis, we generated flies co-expressing Plk4-NG (in a *Plk4* mutant background) and the centriole cartwheel component Sas-6-mCherry, which is irreversibly incorporated into the base of the growing daughter centriole cartwheel and can be used to monitor centriole growth in fly embryos (Aydogan et al., 2018). These flies laid embryos that often failed to hatch (Figure S3C), but we simultaneously measured Plk4 oscillations and centriole growth in those embryos that appeared to be developing normally (Figures 2A, S3A, and S3B; Video S2). The mother centrioles in these embryos were often slightly delayed in initiating daughter centriole growth (Figures 2A, S3D, and S3E), allowing us to measure the amount of Plk4 at the centrioles when daughter centrioles either started or stopped growing (Figure 2A, colored dotted lines).

Video S2. Monitoring Sas-6-mCherry Incorporation and Plk4-NG Oscillations Simultaneously in the Same Embryo, Related to Figure 2 Time-lapse movie of an embryo expressing one copy of Sas-6-mCherry (expressed transgenically from the endogenous *Sas-6* promotor) and two copies of Plk4-NG (expressed transgenically from the endogenous *Plk4* promoter) in a *Plk4* mutant background, observed on a spinning-disk confocal microscope during S-phase of nuclear cycle 12. The movie is a maximum-intensity projection that has been photo-bleach corrected, but not background subtracted for visual clarity. Time (Min:Sec) is shown at the top left, and stage of the cell cycle is indicated at the bottom left.

Strikingly, the centriolar levels of Plk4 at which centriole growth initiated at each cycle (“Start”; Figures 2A and 2B) were not significantly different than the levels at which centriole growth stopped *(*“Stop”; Figures 2A and 2B). This suggests that at each cycle there is a threshold level of centriolar Plk4 that is required to support centriole growth: above this threshold the centrioles can grow, below this threshold they cannot. If the threshold concept is correct, then mother centrioles that failed to recruit sufficient Plk4 should not grow a daughter. We observed that the centrioles in a fraction of the embryos expressing both Plk4-NG and Sas-6-mCherry (mostly at nuclear cycle 13) separated at the start of S-phase but did not detectably incorporate Sas-6-mCherry, indicating that daughter centrioles did not grow (Figures S3D and S3E)—a defect that may explain why many of these embryos failed to hatch (Figure S3C). Intriguingly, centriolar Plk4 levels continued to oscillate in these embryos, but the average amplitude of these oscillations was lower than in the embryos in which centrioles continued to duplicate—and it was almost always below the average threshold at which centriole growth was normally initiated (Figure 2C). Together, these results suggest that the Plk4 oscillations initiate, and determine the duration of, centriole growth.

### Mathematical Modeling of the Plk4 Oscillation

Oscillations in biology are often generated by delayed feedback circuits (Tsai et al., 2008). In *Drosophila*, Plk4 is recruited to centrioles by Asterless (Asl), which also activates Plk4, allowing it to phosphorylate both itself and Asl at multiple sites (Boese et al., 2018, Dzhindzhev et al., 2010, Klebba et al., 2015). Human Asl (Cep152) also binds, and is phosphorylated by, Plk4 *in vitro* (Cizmecioglu et al., 2010, Hatch et al., 2010). We realized that this system could form a time-delayed negative feedback network capable of generating Plk4 oscillations if the activation of Plk4 by Asl eventually led to the inhibition of their interaction.

**Figure 3:**
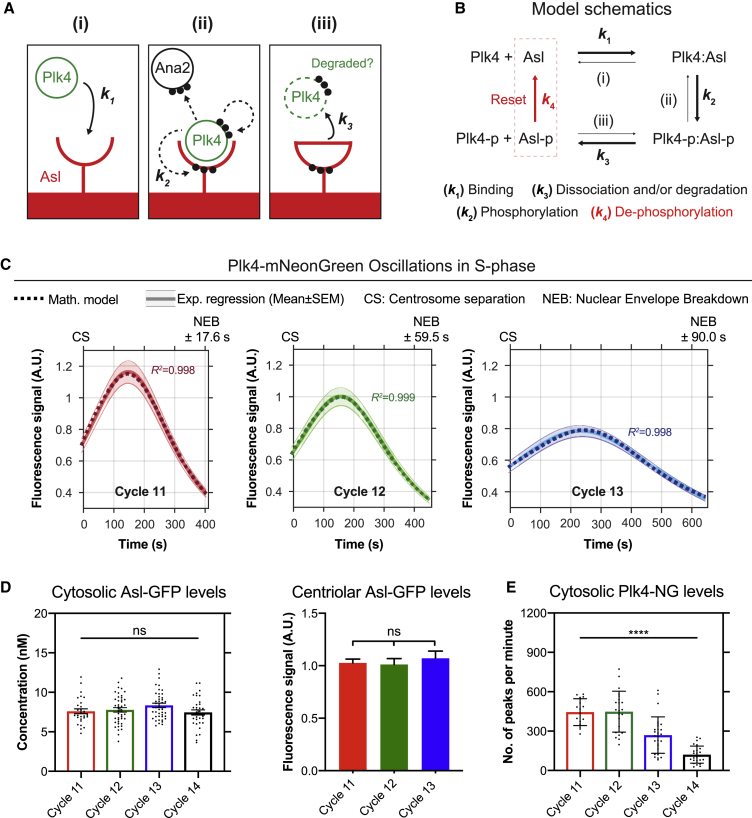
A Simple Mathematical Model of the Plk4 Oscillation and Experimental Investigations to Test Its Predictions (A) Diagram of the model. (i) During mitosis, Asl receptors (red) on the surface of the mother centriole start to bind Plk4 (green) with high affinity (*k*_*1*_). (ii) Once bound, Plk4 is activated, and it starts to phosphorylate itself, Ana2 (black) and Asl (*k*_*2*_) at multiple sites (indicated by dotted black arrows and black dots). (iii) We speculate that, after several rounds of phosphorylation, Asl is converted to a state with low affinity for Plk4, so phosphorylated Plk4 is released (*k*_*3*_)—and likely degraded. These Asl receptors are now inactivated and can no longer bind Plk4 to promote centriole growth. (B) Schematic depicts the topology of the mathematical model (see STAR Methods for full details of the model). Asl-p and Plk4-p indicate phosphorylated proteins. Bold arrows indicate the dominant direction of the reactions. This model discretely examines centriolar Plk4-NG levels only during S-phase of each cycle. We speculate that a phosphatase normally removes the phosphate groups from Asl during mitosis to reset the system for the next oscillation (red arrow; *k*_*4*_), and we extend the model to include this step elsewhere (Figures S4A and S4B). (C) Graphs show the Lorentzian fit of the Plk4-NG oscillation data during S-phase of cycles 11–13 (solid lines) overlaid with analytical solutions to the model (dotted lines). R^2^ values indicate goodness-of-fit. (D) Bar charts quantify the average cytosolic concentration and centriolar fluorescence of Asl-GFP at the start of S-phase of each cycle. Cytosolic Asl-GFP was measured using FCS; each data point represents the average of 4–6 10-s recordings from a single embryo (Figure S5). Centriolar Asl-GFP was measured using confocal microscopy, as described in STAR Methods. N ≥ 14 embryos for each cell cycle; n = 48, 70, and 130 centrioles (mean) per embryo in cycles 11–13, respectively. Data are presented as mean ± SEM. Statistical significance was assessed using an ordinary one-way ANOVA test (ns, not significant). (E) Bar chart shows the relative abundance of Plk4-NG at the start of each nuclear cycle measured by PeCoS (see STAR Methods; Figure S6). Each data point represents a single 180-s recording from a single embryo. Statistical significance was assessed using a Kruskal-Wallis test (^∗∗∗∗^p < 0.0001). Data are presented as mean ± SD. See also Figure S4, Figure S5, Figure S6 and Data S1 (first and fourth charts as well as the Monte Carlo analysis).

A simple version of such a scenario is illustrated in Figures 3A and 3B. At the start of each oscillation cycle, we envisage that unphosphorylated Asl receptors on the mother centriole recruit Plk4 to the site of daughter centriole assembly with high affinity (Figure 3A, (i)). Binding activates Plk4, allowing it to phosphorylate itself (Cunha-Ferreira et al., 2013, Holland et al., 2010, Klebba et al., 2013), Ana2/STIL (Dzhindzhev et al., 2014, Kratz et al., 2015, McLamarrah et al., 2018, Ohta et al., 2014) and Asl/Cep152 (Boese et al., 2018, Hatch et al., 2010) at multiple sites (Figure 3A, (ii)). The phosphorylated Ana2 promotes cartwheel assembly, potentially explaining why a threshold level of Plk4 is required to promote centriole growth—but in our model this reaction is not important for the Plk4 oscillation per se, so we do not consider it further. We speculate that the phosphorylation of Asl at multiple sites reduces its affinity for Plk4, so that the bound Plk4 molecules are released, leaving behind the phosphorylated Asl-receptor that can no longer recruit Plk4 (Figure 3A, (iii)) (see the end of this section for how this network can be reset to trigger subsequent rounds of oscillations).

**Figure S5:**
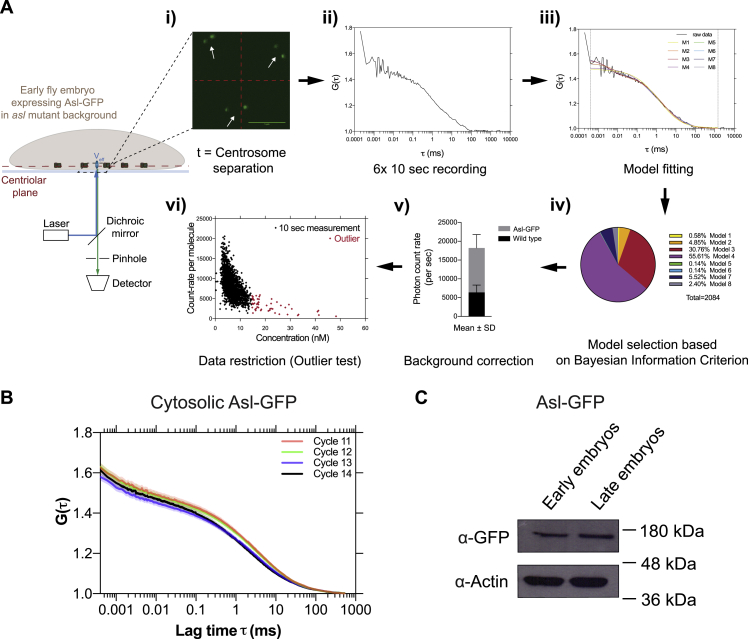
FCS Analysis of Cytosolic Asl Levels, Related to Figure 3 (A) Schematic workflow describes the acquisition and analysis of point Fluorescence Correlation Spectroscopy (FCS) measurements (see STAR Methods for further details). The 488nm laser beam is positioned at the centriolar plane in embryos expressing 2 copies of Asl-GFP (under the control of its own promoter in an *asl* mutant background). (i) At the beginning of every cycle, when the old and new mother centrioles have just separated (*white* arrows), 6x 10 s FCS measurements were taken at a point in the cytosol maximally distant from the centrioles (center of *red* crosshairs). (ii) This generated 6 autocorrelation functions (ACFs) (a typical example is shown here). (iii) In the FoCuS-point software, 8 different models were fitted to each ACF*.* (iv) The model that best fitted the majority of the data (#4 in this case) was chosen based on the Bayesian information criterion, and all ACFs were then fitted to this model. (v) The fitted ACFs were corrected for background noise which was determined by measurements in WT embryos. (vi) The ACFs used for further analysis were then restricted by excluding individual outlier measurements based on a ROUT-outlier test (Q = 1%) (these outlier measurements usually had a poor signal-to-noise ratio and gave concentrations that were often biologically unrealistic, and were presumably generated when a centriole or non-specific fluorescent structure passed through the analyzed volume). (B) Graph shows the average ACFs (represented as Mean ± SEM) for nuclear cycles 11-14 before background corrections. All individual ACFs were used to calculate the cytosolic concentration data shown in Figure 3D. (C) Western blot shows the protein levels of the Asl-GFP in either the early or late cell cycles from embryos of the same genotype used in (A) and (B). This supports the results obtained from the FCS measurements, and suggests that total Asl levels do not change significantly during the development of the syncytial embryo. Early and late embryos were separated based on their distinct morphology (judged by eye using a dissection microscope). Actin is shown as a loading control. A representative blot is shown from two technical repeats.

This network (Figure 3B; see mathematical model 1 in STAR Methods) maps onto a set of coupled linear ordinary differential equations, which we solved analytically. Solutions to this first model (model 1 in STAR Methods) fit the discrete Plk4 oscillation data from each S-phase of nuclear cycles 11–13 very well (Figure 3C; R^2^ > 0.99). Although the model may overfit the data, these solutions were within a reasonable and generally narrow parameter space (Figures 3C and S5; Data S1, first and fourth charts). Nevertheless, we believe this model is likely to be oversimplified. Plk4’s ability to phosphorylate itself, for example, could help to generate the oscillation by promoting Plk4 degradation (Cunha-Ferreira et al., 2009, Guderian et al., 2010, Holland et al., 2010, Rogers et al., 2009) or lowering the affinity of the Asl::Plk4 interaction—as has recently been demonstrated (Park et al., 2019). Moreover, the model considers the behavior of only Asl and Plk4, when other factors, such as Ana2/STIL, are likely to modulate the systems behavior (Arquint and Nigg, 2016, Gönczy and Hatzopoulos, 2019). Finally, the model does not consider the possibility that Plk4 bound to one receptor could phosphorylate nearby receptors, or the Plk4 bound to nearby receptors, to influence their behavior—a concept that may be important when considering how Plk4 ultimately localizes to only a single site on the side of the mother centriole (Leda et al., 2018, Takao et al., 2019).

**Figure S4:**
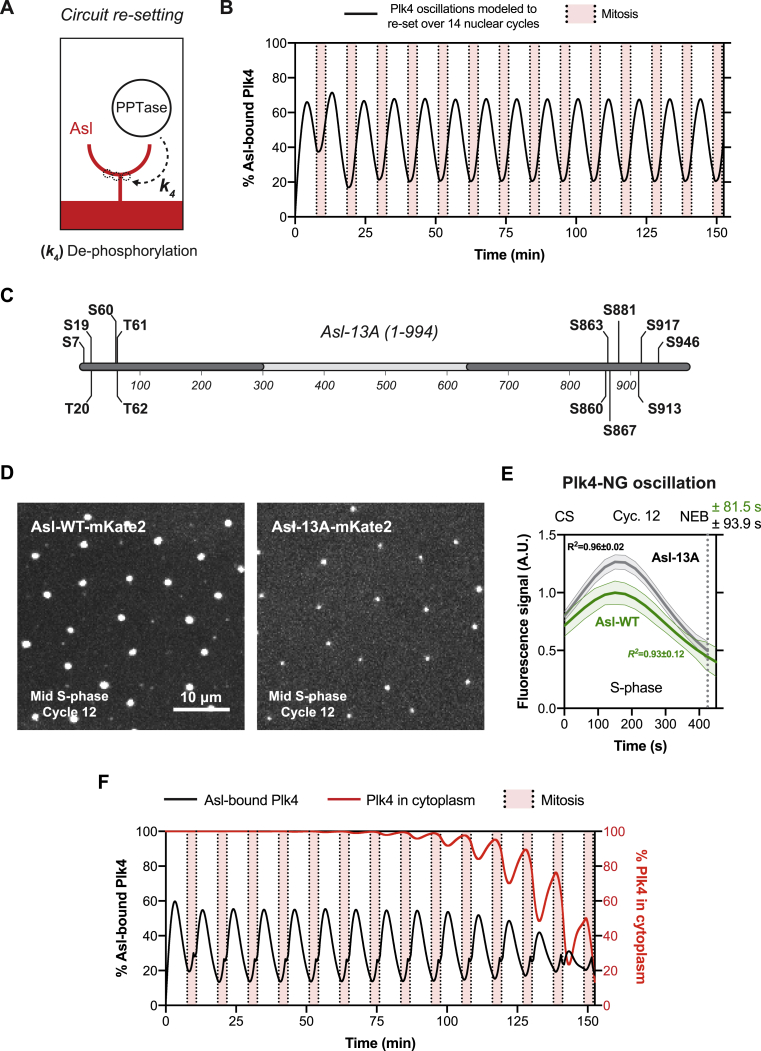
Theoretical and Experimental Assessment of Several Assumptions Made in the Mathematical Model, Related to Figure 3 (A) Our mathematical model depicted in Figures 3A and 3B only discretely examines the Plk4-NG oscillation during S-phase of each nuclear cycle. The schematic here shows our speculation that a phosphatase normally removes the phosphate groups (*dotted* circles) from Asl (*red*) during mitosis to reset the system for the next oscillation at rate *k*_*4*_ (*dotted black* arrow). (B) We implemented this step to extend the original model and plotted the mathematical solution for the percentage of Asl-bound Plk4 molecules (*black* curve) for a total of 14 nuclear cycles. For simplicity we kept the length of S-phase and mitosis constant through all 14 cycles (see STAR Methods for further details of this extended model). (C) Schematic shows the Serine (S) and Threonine (T) residues (in bold) that were mutated to Alanine in the Asl-13A construct. *Dark gray* boxes show the relative positions of the previously mapped Plk4-interacting regions within the N-terminal (Dzhindzhev et al., 2010) and C-terminal (Klebba et al., 2015) regions of Asl. (D) Micrographs show images from time-lapse movies of embryos expressing Asl-WT-mKate2 and Asl-13A-mKate2 (under the control of their own promoters in an *asl* mutant background), respectively. (E) Graphs show the regression data (*solid* lines) for Plk4-NG oscillations in cycle 12 in embryos expressing either Asl-WT (*green*) or Asl-13A (*dark gray*) (both without any fluorescent tag) simultaneously with Plk4-NG. N ≥ 25 embryos for each condition; n = 71 and 68 centrioles (mean) per embryo in *Asl-WT* or *Asl-13A*, respectively (collection of two trials performed by two independent researchers, blinded for each other’s data). Data are presented as Mean ± SEM *R*^*2*^ values indicate goodness-of-fit for the regressions. CS = Centrosome separation; NEB = Nuclear envelope breakdown. (F) In (B) it is assumed that the cytosolic concentration of Plk4 is kept constant over all cycles. The graph here plots an alternative model where the total number of Plk4 molecules in the embryo is kept constant at all cycles. The number of centrioles doubles each cycle, and the mathematical solution for the percentage of Asl-bound Plk4 molecules (*black* curve), and the percentage of Plk4 molecules that remain in the cytoplasm (*red* curve), is depicted over 14 nuclear cycles (see STAR Methods for further details and implications of this model). For the first few nuclear cycles, almost all of the Plk4 remains in the cytoplasm since there are only a few centrioles. In the later cycles, however, the amount of Plk4 sequestered by the Asl receptors increases exponentially, as the number of centrioles increase by a factor of 2 in each cycle. Therefore, the rate at which the Asl receptors are able to recruit Plk4 from the cytoplasm decreases, resulting in a reduction in the amplitude of the Plk4 oscillation. This aspect of the model is consistent with our experimental observations that the amplitude of the Plk4 oscillation decreases at later cycles (Figure 1), as does the cytosolic concentration of Plk4 (Figure 3E). An alternative, or additional, mechanism that might explain these observations is that the Plk4 molecules activated by binding to Asl may be more likely to autophosphorylate to stimulate their degradation, so ensuring that more Plk4 is degraded at each cycle as the number of centrioles increase. Interestingly, in either of these scenarios, increasing centriole numbers leads to increasing Plk4 depletion from the cytosol, potentially allowing embryos to effectively “count” their centrioles.

In order to demonstrate how this network could be reset for the next oscillation, we extended our model (model 2 in STAR Methods) to allow a protein phosphatase (PPTase) to be activated during M-phase to dephosphorylate Asl (Figures 3B, red arrow, and S4A). This resetting is biologically plausible, because the activities of several PPTases are regulated during the cell cycle (Nilsson, 2019). This model can be solved exactly, and its solutions generate robust centriolar Plk4 oscillations within the context of a system that, like the early *Drosophila* embryo, alternates between periods of S- and M-phases (Figure S4B). Thus, our minimal model illustrates that a classical “time delayed negative-feedback” network (Novák and Tyson, 2008) can generate Plk4 oscillations, although the precise molecular details of this system remain to be fully elucidated.

### Testing Predictions of the Mathematical Models

A key feature of our models is that the phosphorylation of Asl by Plk4 reduces their affinity (although, as discussed above, Plk4’s ability to phosphorylate itself, and other factors, could also help to generate the oscillation). To test the plausibility of this idea, we mutated 13 potential Plk4 phosphorylation sites in Asl to Ala (Asl-13A) (Figure S4C). These sites were selected based on their conservation, their similarity to known Plk-family consensus sites (Leung et al., 2007), their proximity to the N- and C-terminal regions of Asl that are thought to interact with Plk4 (Boese et al., 2018), and a previous analysis of sites in the Asl N-terminal region that are either phosphorylated by Plk4 kinase domain *in vitro* or have been shown to be phosphorylated in cultured *Drosophila* cells (Boese et al., 2018). If some of these sites are normally phosphorylated by Plk4 to reduce the affinity of the Asl::Plk4 interaction, we would predict that expressing Asl-13A in the presence of endogenous, unlabeled Asl would lead to an increase in centriolar Plk4-NG levels—because the Plk4 should unbind from the mutant Asl receptors less efficiently. Although Asl-13A-mKate2 localized to centrioles less efficiently than Asl-WT-mKate2 (Figure S4D), expressing untagged Asl-13A increased the amplitude of the Plk4-NG oscillation (Figure S4E), consistent with our idea that phosphorylating Asl can reduce its affinity for Plk4 (Figures 3A and 3B).

**Figure S6:**
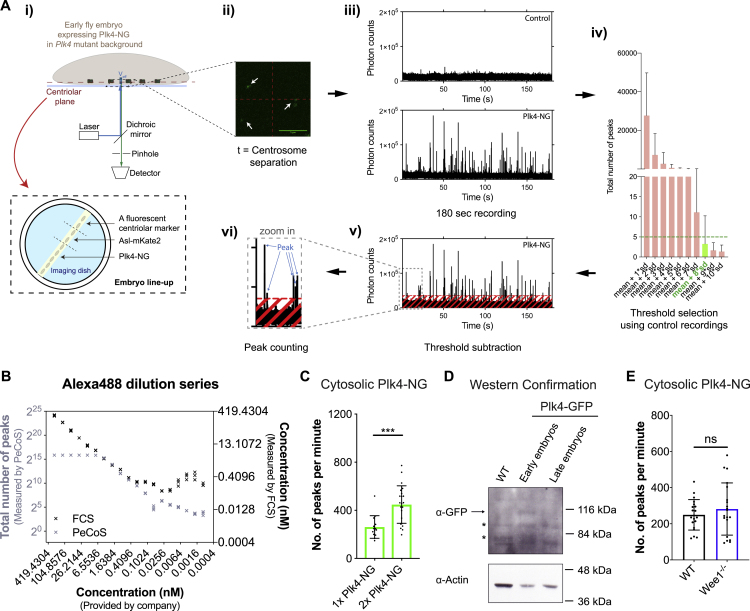
Peak Counting Spectroscopy Analysis of Cytosolic Plk4 Levels, Related to Figures 3 and 6 (A) Schematic workflow describes the acquisition and analysis of *Peak Counting Spectroscopy* (PeCoS) measurements. (i) In addition to embryos expressing Plk4-NG under its own endogenous promoter, embryos of two other genotypes were placed on the same imaging dish. One expressing a green-fluorescent centriole marker to allow correction of the spherical aberration caused by coverslip thickness variation, the other expressing Asl-mKate2 to determine the autofluorescence background threshold for the Plk4-NG expressing embryos—Asl-mKate2 allows one to determine the correct plane (containing the centrioles; *white* arrows) for background measurement, while the mKate2 fluorophore does not interfere with the PeCoS measurements. (ii) As for FCS (see Figure S5), a 488nm laser beam is positioned near the cortex of embryos, and the measurements are taken at a single point in the cytosol (*red* crosshairs) at the beginning of S-phase, but for 1x 180 s, in both control and Plk4-NG expressing embryos (iii). Afterward, (iv) an appropriate threshold is calculated from the control embryos, so that the background contributes less than 5 peaks on average during each recording. Following background subtraction, (v and vi) the number of peaks is quantified. (B) To compare the effective linear concentration range of FCS and PeCoS we assessed a two-fold dilution series of the Alexa488 dye. At high dye concentrations, FCS (*black* symbols) exhibits a near-linear response, while PeCoS (*gray* symbols) is saturated—presumably because there are too many fluorophores in the effective volume (*V*_*eff*_) for them to be measured as individual peaks. At intermediate dye concentrations, both methods exhibit a near linear response. At low concentrations (~ < 0.2nM), however, FCS becomes unreliable while PeCoS continues to have a near-linear response. (C) The bar chart shows the *in vivo* validation of PeCoS. A significant difference in the number of peaks per minute was observed between embryos expressing either 1x or 2x copies of Plk4-NG (under the control of its endogenous promoter), which were measured at the beginning of S-phase in nuclear cycle 12. Each data point represents a 180 s recording from a single embryo. Statistical significance was assessed using Mann-Whitney test (^∗∗∗^p < 0.001). Data are presented as Mean ± SD (D) Western blot analysis of Plk4-GFP (arrow) levels in early and late embryos supports the conclusion from the PeCoS analysis (Figure 3E) that cytosolic Plk4 levels are lower in late embryos than in early embryos. Prominent non-specific bands are indicated (^∗^). A representative blot is shown from two technical repeats. (E) The bar chart compares the cytosolic levels of Plk4-NG (under the control of its endogenous promoter; at the beginning of S-phase in Cycle 13) between WT and *Wee1*^*−/−*^ embryos (the same genotypes as in Figure 6). Statistical significance was assessed using an ordinary unpaired t test (ns, not significant). Data are presented as Mean ± SD.

An inspection of the parameters generated by our model revealed that the reduction in the amplitude of the Plk4 oscillation at successive nuclear cycles was driven primarily by a reduction in the cytosolic concentration of Plk4 (that determines *k*_1_, the rate at which Plk4 binds to Asl), while total levels of the Asl receptor (*A*_*tot*_) remain relatively constant (Data S1, first chart). To test if this was the case, we first used fluorescence correlation spectroscopy (FCS) (Figure S5) to examine the cytosolic concentration of Asl-GFP. Although the number of centrioles assembled doubles at each successive cycle, the average cytosolic concentration of Asl-GFP, and the average centriolar levels of Asl-GFP, remained relatively constant at the start of each successive cycle (Figure 3D), as predicted by our model. Unfortunately, the cytosolic concentration of Plk4-NG was too low to be measured by conventional FCS, so we developed a new method, peak counting spectroscopy (PeCoS), to measure relative protein abundance at lower concentrations (see STAR Methods) (Figure S6). This revealed that, in contrast to Asl-GFP, the cytosolic levels of Plk4-NG tended to decrease at successive nuclear cycles (Figure 3E), as predicted by the model.

Why do cytosolic Plk4 levels decrease at successive nuclear cycles? Our modeling suggests that if total Plk4 levels in the developing embryo remain constant (i.e., the rate of Plk4 degradation and synthesis are balanced), then the doubling of centriole numbers at each cycle can lead to the depletion of cytosolic Plk4—particularly during later nuclear cycles—as an increasing fraction of the protein is sequestered by the increasing number of centrioles (Figure S4F). Alternatively (or additionally), Plk4 molecules that are activated by binding to Asl may be more likely to phosphorylate themselves to stimulate their degradation, ensuring that more Plk4 is degraded at each cycle as the number of centrioles increase. Interestingly, in either of these scenarios, increasing centriole numbers lead to more Plk4 depletion from the cytosol, potentially allowing embryos to effectively “count” their centrioles.

### The Plk4 Oscillation Can Adapt to Changes in Plk4 Levels to Maintain a Constant Centriole Size

**Figure 4:**
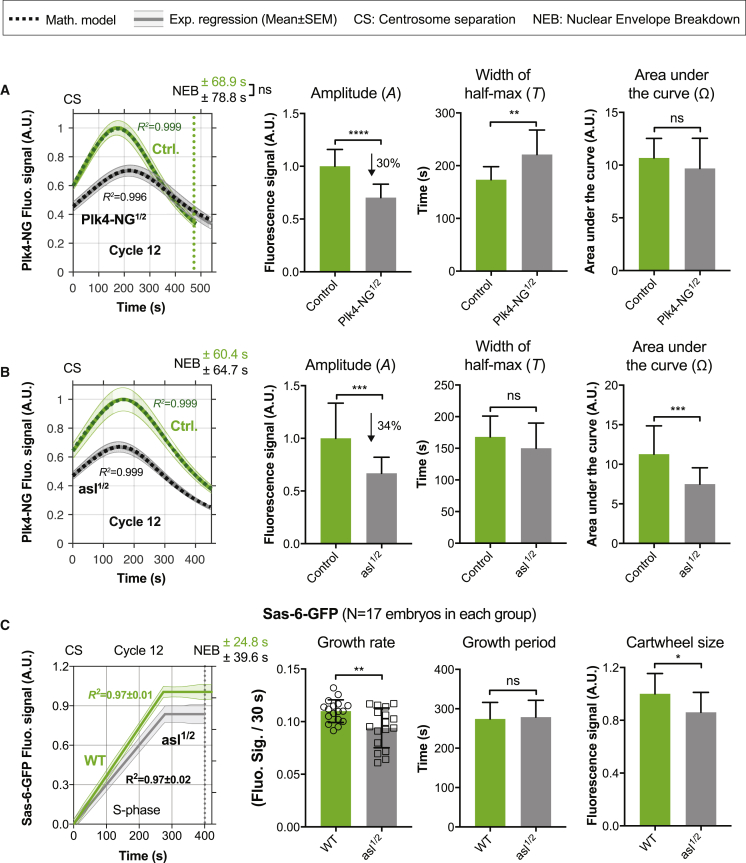
The Plk4 Oscillator Can Adapt to Changes in Plk4 Concentration but Not to Changes in Asl Concentration (A and B) Graphs show the regression data (solid lines) and mathematical solutions (dotted lines) for Plk4-NG oscillations in cycle 12 for experiments where either (A) the genetic dose of *Plk4-NG* was halved (*Plk4-NG*^*1/2*^), or (B) the genetic dose of *asl* was halved (*asl*^*1/2*^) (gray lines) compared to controls (green lines). (A) N ≥ 11 embryos for each condition; n = 47 and 42 centrioles (mean) per embryo in control or *Plk4-NG*^*1/2*^ groups, respectively. (B) N = 18 embryos for each condition; n = 44 and 43 centrioles (mean) per embryo in control or *asl*^*1/2*^ groups, respectively. Data are presented as mean ± SEM. Bar charts quantify oscillation parameters, as indicated; data are presented as mean ± SD. (C) Graph quantifies the parameters of cartwheel growth—as measured by Sas-6-GFP fluorescence incorporation (Aydogan et al., 2018)—in WT and *asl*^*1/2*^ embryos; data are presented as mean ± SEM. Bar charts quantify growth parameters presented as mean ± SD. N = 17 embryos for each condition; n = 77 and 72 centrioles (mean) per embryo in WT or *asl*^*1/2*^ groups, respectively. Statistical significance was assessed using an unpaired t test with Welch’s correction (for Gaussian-distributed data) or an unpaired Mann-Whitney test (^∗^p < 0.05; ^∗∗^p < 0.01; ^∗∗∗^p < 0.001; ^∗∗∗∗^p < 0.0001; ns, not significant). R^2^ values indicate goodness-of-fit for the mathematical solutions. See also Figure S7 and Data S1 (second and third charts).

**Figure S7:**
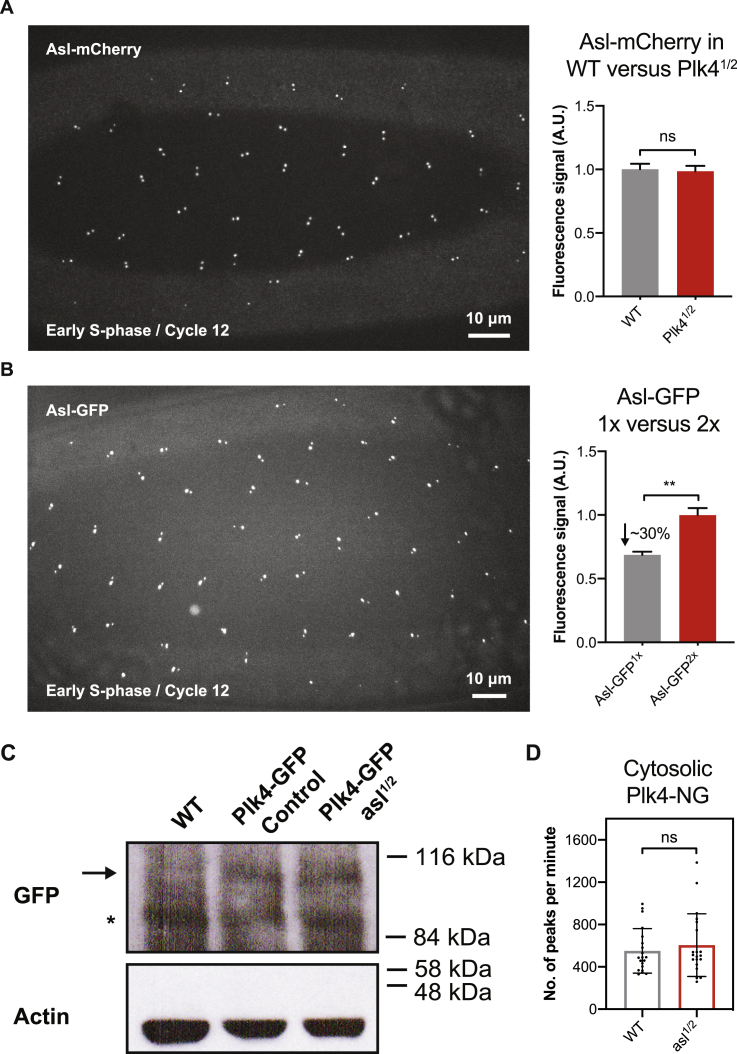
Quantification of Centriolar Asl and Cytosolic Plk4 Levels When the Genetic Dose of *asl* or *Plk4* Is Halved, Related to Figure 4 (A) Micrograph shows an image of Asl-mCherry at centrioles in an embryo in early S-phase (just after centrosome separation). Bar charts quantify the average centriolar Asl-mCherry levels in early S-phase in either WT embryos (WT) or in embryos where the genetic dose of *Plk4* has been halved (*Plk4*^*1/2*^). N = 17 embryos for each condition; n = 67 and 58 centrioles (mean) per embryo in WT or *Plk4*^*1/2*^ groups, respectively. Average centriolar Asl levels do not change significantly when the genetic dosage of *Plk4* is halved, in agreement with the prediction of our model (see Data S1; first, second and third charts). (B) Same schema as (A), but showing the localization of Asl-GFP, and quantifying the centriolar levels of Asl-GFP in *asl* mutant embryos expressing either 1 (Asl-GFP^1x^) or 2 (Asl-GFP^2x^) copies of Asl-GFP. N = 10 embryos for each condition; n = 59 and 54 centrioles (mean) per embryo in Asl-GFP^1x^ or Asl-GFP^2x^ groups, respectively. This analysis reveals that centriolar Asl-GFP levels drop by ~30% when the genetic dosage of *Asl-GFP* is halved, in good agreement with the prediction of our model (see Data S1; first, second and third charts). Data are represented as Mean ± SEM. Statistical significance was assessed using an unpaired t test with Welch’s correction (for Gaussian-distributed data) or an unpaired Mann-Whitney test (^∗∗^p < 0.01; ns, not significant). (C) Western blot compares the protein levels of Plk4-GFP (*arrow*) (expressed under the control of its own promoter in a *Plk4* mutant background) in otherwise WT embryos or in embryos in which the genetic dosage of *asl* has been halved. This analysis reveals that Plk4-GFP levels in the embryo do not change dramatically when the genetic dosage of *asl* is halved, in agreement with the prediction of our model. WT embryos (Lane 1) are shown as a negative control to demonstrate that the Plk4-GFP band is only detected in embryos expressing Plk4-GFP. Prominent non-specific bands are indicated (^∗^). Actin is shown as a loading control. A representative blot is shown from two technical repeats. (D) The bar chart compares the number of Plk4-NG peaks per minute that was observed between normal embryos (*WT*) or embryos where the genetic dose of Asl was halved (*asl*^*1/2*^). Measurements were performed at the beginning of S-phase in nuclear cycle 12. Each data point represents a 180 s recording from a single embryo. Statistical significance was assessed using an ordinary unpaired t test (for Gaussian-distributed data) or a Mann-Whitney test (ns, p > 0.05). Data are presented as Mean ± SD.

Our finding that cytosolic levels of Asl remain constant at successive cycles while cytosolic Plk4 levels decrease suggests a rationale for why centriole biogenesis may be regulated by an oscillatory system. In our models, Asl effectively functions as an integrator (Ferrell, 2016, Somvanshi et al., 2015) whose levels are kept constant so that it can measure changes in the input (cytosolic Plk4 levels) and adapt the oscillation to maintain a constant output (centriole size). If this interpretation is correct, then the Plk4 oscillation should adapt to maintain a constant centriole size when Plk4 levels change, but not when Asl levels change. To test this, we monitored Plk4-NG oscillations in embryos laid by mothers where we genetically halved the dose of either Plk4-NG (hereafter *Plk4-NG*^*1/2*^ embryos) or *asl* (hereafter *asl*^*1/2*^ embryos). Centrioles appeared to duplicate normally in both sets of embryos, but the Plk4 oscillation parameters were altered: in *Plk4-NG*^*1/2*^ embryos, *A* decreased but there was a compensatory increase in *T*, so Ω remained relatively constant (Figures 4A and S7A); in *asl*^*1/2*^ embryos, *A* decreased, but there was no compensatory change in *T*, so Ω decreased (Figures 4B and S7B–S7D).

Our mathematical model (model 1) could fit both sets of data well (Figures 4A and 4B; R^2^ > 0.99), generating a reasonable range of parameters (Data S1, second and third charts), several of which we again validated experimentally (Figure S7; see mathematical modeling section in STAR Methods). Interestingly, if we took the normal parameters derived from our model and simply adjusted the amount of Asl or Plk4 in the model to the levels we experimentally measured in the half-dose embryos, the model fit the data less well (not shown). This suggests that changing the concentration of one component is likely to influence the concentration and/or behavior of other components so that several parameters of the Plk4 oscillation are altered. This seems plausible, as the core centriole duplication proteins are known to interact with and influence each other in multiple ways (Arquint and Nigg, 2016, Gönczy and Hatzopoulos, 2019, Nigg and Holland, 2018).

Consistent with our observation that the Plk4-NG oscillations adapt in *Plk4-NG*^*1/2*^ embryos by reducing *A* and increasing *T* to maintain a relatively constant Ω, we previously showed that halving the genetic dose of Plk4 led to the centrioles growing slowly, but for a longer period of time, to maintain a constant size (Aydogan et al., 2018). In contrast, we would predict that daughter centrioles in *asl*^*1/2*^ embryos should grow more slowly (as *A* is decreased), but for a normal period (as *T* is unchanged), and so centrioles would be too short (as Ω decreases). We measured the parameters of daughter centriole growth in *asl*^*1/2*^ embryos and confirmed that this was the case (Figure 4C). Together, these experiments suggest that the Plk4 oscillatory network functions to maintain a constant centriole size even when Plk4 levels vary.

### Plk4 Oscillations Can Execute Centriole Duplication Independently of a Robust Cdk/Cyclin Cell-Cycle Oscillator

**Figure 5:**
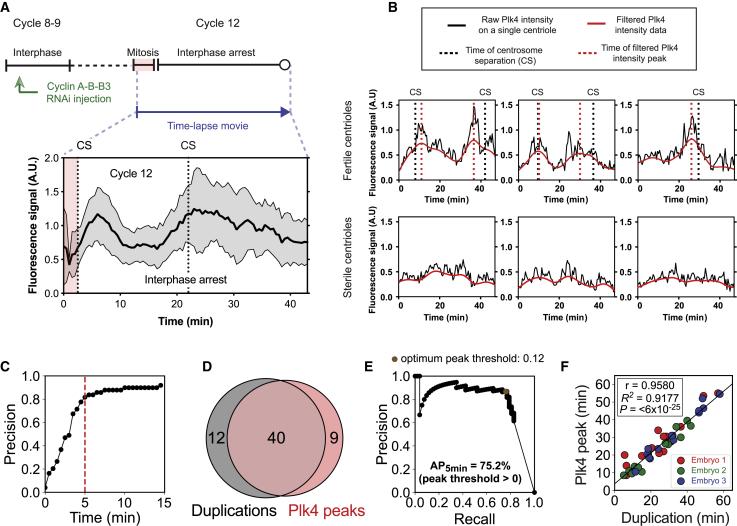
Plk4 Levels Can Continue to Oscillate and Promote Centriole Duplication Even When the CCO is Perturbed (A) Graph shows the Plk4-NG oscillations in an embryo injected with dsRNA against cyclin A-B-B3; the schema above the graph illustrates the experimental protocol. The nuclei in this embryo arrest in interphase, but centrioles go through an additional round of division—centriole separation (CS)—accompanied by a Plk4 oscillation. See Figure S2B for additional examples; n = 30 centrioles (mean) per embryo. (B) Graphs show the raw (black lines) and filtered (red lines) fluorescence intensity data of 3 individual “fertile” centrioles and 3 individual “sterile” centrioles within the same cyclin-depleted embryo. The fertile centrioles duplicate (black dotted lines), and these events were often closely associated with computed Plk4 oscillation peaks (red dotted lines) (see STAR Methods for further details of the peak calling methodology). (C) An unbiased computational analysis of all 45 fertile centrioles in 3 embryos reveals that >80% of the computationally detected Plk4 oscillation peaks occur within 5 min of an experimentally observed duplication event. A simulation with randomly distributed centriole duplication events and Plk4 oscillation peaks showed a mean time separation of 10.5 min (data not shown). (D) Venn diagram shows how, using a 5-min window, the oscillation peaks can be used to predict duplication events with both high precision and high recall (40/49 Plk4 oscillation peaks are associated with a duplication event, and 40/52 duplication events are associated with a Plk4 oscillation peak). (E) Graph shows the ability of Plk4 oscillation peaks to “retrieve” centriole duplication events across all peak prominences. All detected oscillation peaks were ranked in order of their peak prominence from high to low (black dots) and assigned uniquely to a duplication event if within a 5-min time window. The graph then plots the precision and recall values if the threshold for calling a peak were set as the peak prominence value of each peak (in descending order). Below the detected peak that is associated with a peak prominence threshold of 0.12, the precision dramatically drops, suggesting the existence of a minimum peak amplitude for centriole duplication. At this threshold, precision and recall are jointly optimized. Note, if there were no overall correlation between Plk4 peaks and a duplication event, the integrated area under the curve across all peak prominences or average precision (AP) for the 5-min time window (AP_5min_) would be ~50% (given by # duplications/(# duplications + # peaks)); so the score of ~75% indicates a meaningful correlation. (F) Graph shows the correlation between the time of the computationally determined Plk4 peaks and their respective experimentally observed duplication events. Correlation strength was examined using Pearson’s correlation coefficient (r < 0.40 weak; 0.40 < r < 0.60 moderate; r > 0.60 strong); significance of correlation was determined by the p value (p < 0.05) (see STAR Methods for a full description of this analysis). See also Figures S2 and S8.

Although the Plk4 oscillations in fly embryos are normally entrained by the cell-cycle oscillator (CCO) (Figures 1E and 1F), it has long been known that centrioles can continue to duplicate in many systems even when several other aspects of cell-cycle progression are blocked (Balczon et al., 1995, Gard et al., 1990, Sluder et al., 1990). We wondered whether this might be because Plk4 oscillations can continue to drive centriole biogenesis even in the absence of a robust CCO. To test this possibility, we injected embryos with double-stranded RNAs (dsRNAs) targeting the three embryonic mitotic cyclins: A, B, and B3. These embryos arrest in an interphase-like state with intact nuclei that do not duplicate their DNA, but where centrosomes can continue to duplicate (McCleland and O’Farrell, 2008). We initially injected embryos in nuclear cycles 7–8 and monitored Plk4-NG behavior ∼30 min later. In all such embryos, we observed an initial synchronous round of centriole duplication without NEB (indicating that the CCO was perturbed), followed by one or more rounds of less synchronous centriole duplication (Figures 5A and S2B; Video S3). Strikingly, a normal Plk4-NG oscillation was associated with the first, synchronous, round of centriole duplication, but subsequent oscillations were more variable (Figures 5A and S2B).

Video S3. Centrioles Can Continue to Duplicate in Embryos Arrested for a Short Period in Interphase by Mitotic Cyclin Depletion, Related to Figure 5 Time-lapse movie of an embryo expressing two copies of Plk4-NG (expressed transgenically from the endogenous *Plk4* promoter) in a *Plk4* mutant background, observed on a spinning-disk confocal microscope through nuclear cycle 12. The embryo was injected with cyclin A-B-B3 dsRNA in ∼cycle 8, approximately 30-40 min prior to the start of the movie. The movie on the left is the maximum intensity projection of the slices where centrioles are in focus. The movie on the right is the maximum intensity projection of the slices where nuclei are in focus. Note how the centrioles undergo at least two rounds of duplication, the second of which is more asynchronous and occurs without nuclear envelope breakdown (indicating that the nuclei are arrested in an interphase-like state). These videos have been photo-bleach corrected, but not background subtracted for visual clarity. Time (Min:Sec) is shown at the top left.

**Figure S8:**
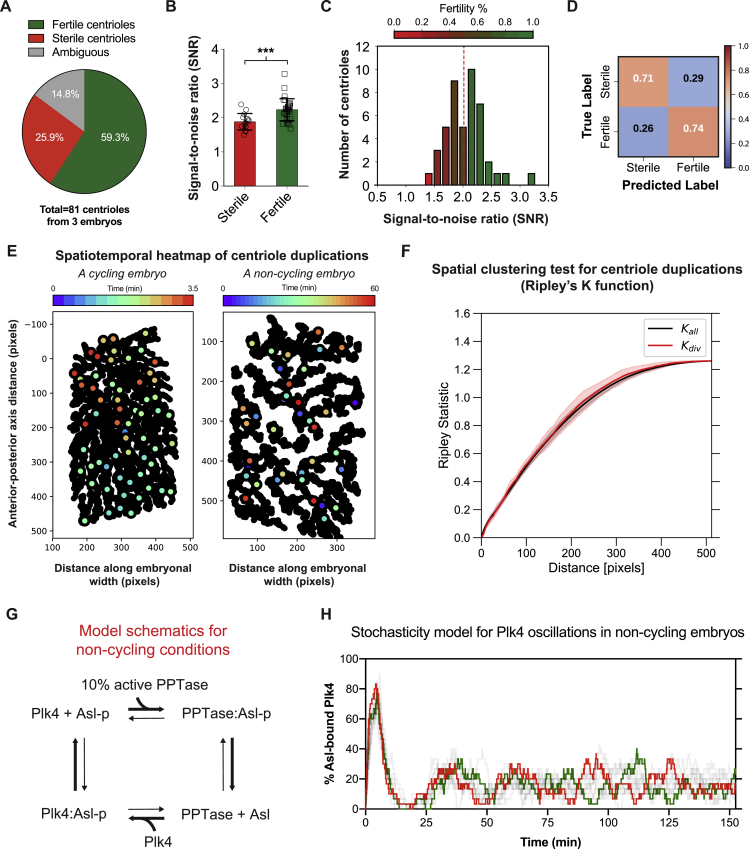
The Average Centriolar Plk4-NG Level on Individual Centrioles Can Be Used to Predict Stochastic Centriole Duplications in Embryos Arrested in Interphase by Mitotic Cyclin Depletion, Related to Figures 5 and S2 (A) The pie chart quantifies the percentage of centrioles that continued to duplicate in embryos where cyclin A-B-B3 dsRNA was injected into embryos at nuclear cycle 2-4, and centriole behavior assessed ~90 min later. *Ambiguous* (gray) indicates the fraction of centrioles whose duplication state could not be unambiguously determined due to their drifting out of focus during imaging. (B) Bar chart shows the mean signal-to-noise ratio (SNR) of Plk4-NG fluorescence signals from sterile and fertile centrioles (*red* and *green*, respectively) through the entire period of observation. Data are presented as Mean ± SD. Statistical significance of SNR was tested using a t test assuming equal variance (^∗∗∗^p < 0.001). (C) Heatmap histogram of all SNR values from sterile and fertile centrioles. *Red dashed* line shows the unbiased threshold, determined automatically from Otsu thresholding for distinguishing sterile and fertile centrioles. Heatmap (*Red*: Sterile and *Green*: Fertile) indicates the fraction of fertile/sterile centrioles in each column. Note that, the higher the SNR, the more fertile the centrioles are. (D) Confusion matrix shows the classification performance of sterile versus fertile centriole Plk4-NG signals using the Otsu threshold in (C) as a proportion of the total number of signals, n = 81 centrioles from 3 embryos. (E) Heatmap plots demonstrate the spatial (x,y) coordinates of all centriole duplication events in a representative cycling embryo (left; at the beginning of cycle 13 when centrioles are separating over the course of ~3.5 min) and non-cycling embryo (right; captured over ~60 min), as each duplication event colored *light blue* to *dark red* to represent early and late time points, respectively. The black points plot the observed spatial (x,y) positions of all centrioles, duplicating and non-duplicating, at all time points. Note that the duplications in the cycling embryo are spatially and temporally coordinated (tending to divide first at the top of the embryo and later at the bottom of the embryo), while the duplications in the non-cycling embryo occur over a longer time-scale and do not appear to be coordinated in space or time. (F) To test more rigorously whether the centriole duplication events in non-cycling embryos are largely stochastic, we calculated Ripley *K* statistics for all the non-cycling embryos used in (A–D). This statistic provides a measure of whether the temporal duplication events have spatial preference by measuring the average number of events that occur as a function of distance from individual centrioles. Curves were computed from the (x,y) coordinates of only the duplicating centrioles (denoted $K_{divpoint}$, *red* line) and of all centrioles at all times (denoted $K_{allpoints}$, *black* line). The sigmoidal increase in the statistic as a function of distance in both cases suggests that duplication events do not cluster spatially at short distances (< 50-100 pixels). The trend and amplitudes of *red* and *black* lines (mean ± SD) are very similar and fall in each other’s statistical confidence range, indicating that duplicating centrioles do not exhibit additional spatial clustering above the natural spatial distribution of centrioles. (G) Schematic depicts the topology of the mathematical model that illustrates how Plk4 oscillations at individual centrioles could be generated to trigger stochastic duplications in non-cycling embryos. Briefly, we no longer assume that a PPTase acts in discrete bursts during mitosis and instead assume a continuous low-level PPTase activity (10% of the activity in cycling embryos). We allow individual Asl receptors to bind Plk4 and be phosphorylated until they release Plk4 (as in our original model), and to be continuously be slowly dephosphorylated by the PPTase. Asl-p and Plk4-p indicate phosphorylated proteins. Bold arrows indicate the dominant direction of the reactions. (H) Graph shows how the percentage of Asl receptors that are bound to Plk4 changes over time at 10 individual centrioles, two of which have been colored *red* or *green*. The centrioles are initially synchronized, as their Asl receptors all start in a dephosphorylated, unbound, state and so exhibit a coordinated pulse of Plk4 binding. As time progresses, however, the Asl receptors lose synchrony (as their dephosphorylation is no longer entrained by the CCO), and so each centriole exhibits low amplitude stochastic oscillations. These oscillations may be sufficient to trigger centriole duplication under these conditions of interphase arrest with low CCO activity, as evident from our experimental observations (Figures 5B–5F and Video S4; see STAR Methods for full details of the model).

We reasoned that any residual Plk4-NG oscillations in these embryos might be triggered by residual CCO oscillations that could trigger centriole duplication, but not DNA synthesis or NEB. While one can never rule out the possibility of residual CCO activity, we tried to overcome this potential problem by examining centriole behavior in embryos in which the CCO was likely to be more fully suppressed by injecting the embryos earlier (nuclear cycles 2–4) and monitoring them later (after ∼90 min). The centrioles in these embryos were now completely dissociated from the non-dividing nuclei and they appeared to divide stochastically, with some centrioles duplicating one or more times, and others not duplicating at all (Figure S8; Video S4). The CCO coordinates cell-cycle events in normal early embryos by spreading as a chemical trigger wave (Chang and Ferrell, 2013, Deneke et al., 2016), but duplicating centrioles did not detectably trigger the duplication of nearby centrioles (Figure S8F). Thus, the “decision” to duplicate in these CCO-suppressed embryos appears to be largely intrinsic to each individual centriole.

Video S4. Centrioles Duplicate Stochastically in Embryos Arrested for a Long Period in Interphase by Mitotic Cyclin Depletion, Related to Figure 5 Time-lapse movie of an embryo expressing two copies of Plk4-NG (expressed transgenically from the endogenous *Plk4* promoter) in a *Plk4* mutant background, observed on a spinning-disk confocal microscope. The embryo was injected with cyclin A-B-B3 dsRNA in ∼cycle 2-4, approximately 90 min prior to the start of the movie. The movie on the left is the maximum intensity projection of the slices where centrioles are in focus. The movie on the right is the maximum intensity projection of the slices where nuclei are in focus. Note that a small number of large nuclei are present throughout the time-course of the movie (indicating that they are arrested in an interphase-like state), but some centrioles duplicate one or more times in an apparently stochastic manner. The Plk4-NG oscillations on individual centrioles are less obvious than in normally cycling embryos, but an unbiased computational analysis of these movies indicates that individual centriole duplication events are correlated with individual centriolar Plk4-NG oscillations (Figures 5B–5F and S8A–S8D). These videos have been photo-bleach corrected, but not background subtracted for visual clarity. Time (Min:Sec) is shown at the top left.

To test whether these stochastic centriole duplications were triggered by Plk4 oscillations, we measured Plk4-NG fluorescence levels at individual centrioles. The raw intensity data were noisy, but duplicating “fertile” centrioles appeared to exhibit more prominent Plk4-NG oscillations than non-duplicating “sterile” centrioles (Figure 5B). Moreover, the average centriolar Plk4-NG fluorescence level (expressed as signal-to-noise ratio [SNR]) was significantly higher at fertile centrioles (Figure S8B), and Plk4-NG SNR values could distinguish fertile and sterile centrioles, correctly predicting centriole fertility or sterility ∼74% and ∼71% of the time, respectively (Figures S8C and S8D).

Upon filtering the raw oscillation data, we found that the peaks of the Plk4-NG oscillations (see STAR Methods for a description of peak-calling methodology) were often associated with centriole duplication events (Figure 5B). An unbiased computational analysis of all the 45 fertile centrioles that we observed in 3 different embryos revealed that the predicted Plk4-NG oscillation peaks predicted centriole duplication events with high precision (40/49 Plk4-NG peaks were associated with a duplication event that occurred within ±5 min of the peak) and recall (40/52 duplication events occurred within ±5 min of a Plk4-NG oscillation peak) (Figures 5C and 5D). Computer simulations revealed that a random distribution of the duplication events lead to an average time of >10 min between the peaks and duplication events, indicating that the observed association was not random. Moreover, a rank ordering of the Plk4-NG oscillations based on amplitude revealed that the higher the amplitude of the oscillation, the more likely it was to be associated with a centriole duplication event (Figure 5E), while plotting the relative timing of the Plk4-NG oscillations and the centriole duplication events revealed a strong positive correlation (Figure 5F; Pearson *r* = 0.9580, p < 0.0001). We conclude that individual centrioles can organize autonomous Plk4 oscillations that can drive centriole duplication even in the absence of a robust CCO. This potentially explains how centrioles can continue to duplicate independently of many other cell-cycle events.

### The CCO Can Phase-Lock the Plk4 Oscillation to Coordinate Centriole Duplication with Other Cell-Cycle Events

It is widely believed that the CCO acts primarily as a “ratchet” whose activity increases over the cell cycle to trigger the sequential execution of cell-cycle events such as DNA replication, centriole duplication, nuclear envelope breakdown (NEB), and spindle assembly (Stern and Nurse, 1996, Swaffer et al., 2016, Swaffer et al., 2018). An interesting alternative possibility is that the CCO could act as a “phase-locker” whose function is simply to entrain the phase of a network of autonomous oscillations, each of which is responsible for the execution of a specific cell-cycle event (Lu and Cross, 2010). The Plk4 oscillation appears to time and execute centriole biogenesis, and it can trigger centriole duplication independently of a robust CCO, so it is an excellent candidate for such an autonomous oscillation.

To better understand how the CCO might entrain the Plk4 oscillation, we measured the average period of the stochastic Plk4 oscillations in cyclin-depleted embryos (20.5 ± 4.6 min) and compared this to the average period of the Plk4 oscillations in cycles 11–12 (11.7 ± 0.7 min) and 12–13 (14.9 ± 1.7 min). The natural period of the autonomous Plk4 oscillation in these early embryos is therefore similar to, but slightly slower than, the period of the Plk4 oscillations normally enforced by the CCO, indicating that the CCO could entrain the Plk4 oscillation by speeding up a phase of its natural cycle.

**Figure 6:**
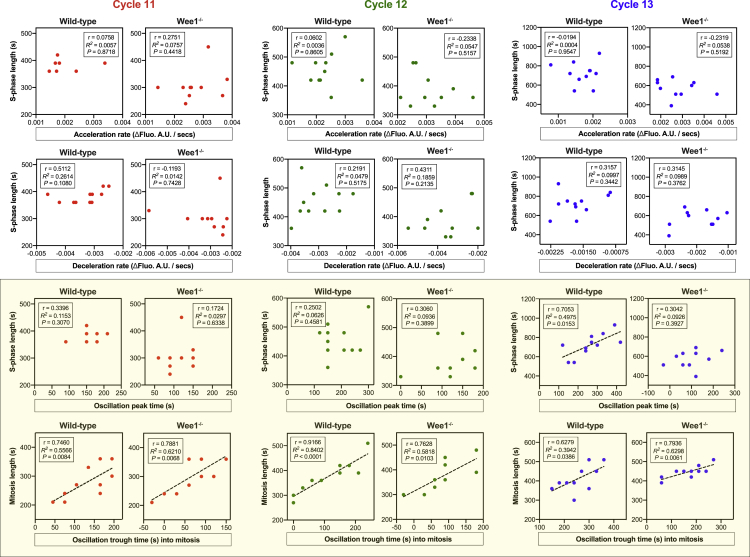
The CCO Phase-Locks the Plk4 Oscillations in Mitosis of Cycles 11–13 Independently of Wee1 and in Interphase of Cycle 13 in a Wee1-Dependent Manner Scatterplots illustrate correlations between various parameters of the Plk4 oscillation and the length of S- or M-phase in nuclear cycles 11–13 in WT and *Wee1*^−/−^ embryos (see Plk4-NG smooth curve fitting and parameter extraction in STAR Methods for details of how these parameters [along with their descriptions] were obtained in an unbiased way). During cycles 11–13, there is a significant correlation between the timing of the Plk4 oscillation trough in M-phase and the duration of M-phase (lower scatterplots in the light yellow panel), suggesting that the CCO entrains the Plk4 oscillation during M-phase. This entrainment is not altered in the *Wee1*^−/−^ embryos. During nuclear cycle 13, there is an additional correlation between the peak of the Plk4 oscillation and S-phase length that is lost in the *Wee1*^−/−^ embryos (upper rightmost scatterplot in the light yellow panel). The plots for the WT group were generated with the data obtained from Figures 1A and S2A, as well as 5 additional embryos of the same genotype. N = 10 embryos; n = 23 centrioles (mean; starting from cycle 11) per embryo in *Wee1*^−/−^ group. Correlation strength was examined using Pearson’s correlation coefficient (r < 0.40 weak; 0.40 < r < 0.60 moderate; r > 0.60 strong); significance of correlation was determined by the p value (p < 0.05). See also Figures S4 and S6E.

To examine which phase this might be, we tested for correlations between various parameters of the Plk4 oscillation and the length of S- or M-phase. During cycles 11–13, we observed a significant correlation between the timing of the Plk4-NG oscillation trough in M-phase and the duration of M-phase (Figure 6, lower scatterplots in the light yellow panel), suggesting that the CCO entrains the Plk4 oscillation by speeding it up during M-phase. This is consistent with our minimal model, in which the CCO entrains the Plk4 oscillation by ensuring the rapid and coordinated dephosphorylation of Asl during M-phase (Figures S4A and S4B).

We also noticed an additional correlation between the peak of the Plk4-NG oscillation and S-phase length in cycle 13 (Figure 6, upper rightmost scatterplot in the light yellow panel). This is not surprising, as a Wee1-dependent checkpoint dramatically slows the CCO—and many other aspects of S-phase progression—particularly during nuclear cycle 13 (Deneke et al., 2016, Stumpff et al., 2004). Moreover, in *Wee1*^−/−^ embryos, the correlation between the Plk4-NG oscillation trough and M-phase length was maintained (Figure 6, lower rightmost scatterplot in the light yellow panel), while the correlation between the Plk4-NG oscillation peak and S-phase length was lost (Figure 6; upper rightmost scatterplot in the light yellow panel), demonstrating that Wee1 can influence the Plk4 oscillation in S-phase. Interestingly, the cytosolic levels of Plk4-NG were essentially the same in wild-type (WT) and *Wee1*^−/−^ embryos (Figure S6E), indicating that cell-cycle regulators can influence the Plk4 oscillation without changing Plk4’s cytosolic concentration. This supports the model prediction that the drop in cytosolic Plk4 levels at successive nuclear cycles (Figure 3E) is not, on its own, sufficient to account for the change in Plk4 oscillation parameters we observe from cycles 11–13. This presumably explains why the model requires several parameters to change slightly at each successive cycle to best fit the data (Data S1, first chart).

Taken together, our observations are consistent with the phase-locker model of cell-cycle regulation (Lu and Cross, 2010). We propose that the Plk4 oscillation may be an exemplar of an autonomously oscillating system that can independently drive a cellular event (centriole duplication), but that is normally phase-locked by the CCO to ensure its proper coordination with other biological events and with cell division.

### A Model to Generate Autonomous Plk4 Oscillations in the Absence of a CCO

How can a Plk4 oscillation be generated independently of the CCO? Our mathematical model (model 2 in STAR Methods) cannot explain this, as it requires a PPTase to reset the system specifically during M-phase (Figures S4A and S4B). Interestingly, if we extend the model to allow the PPTase to have a constant low-level of activity (∼10% of the level normally required to reset the system in M-phase) (Figure S8G) this new model (model 3 in STAR Methods) recapitulates several features of centriole duplication in the cyclin-depleted embryos (Figure S8H). This model predicts that after a last round of mitosis the centrioles in the cyclin-depleted embryos will undergo a single synchronous Plk4 oscillation (as all of the Asl receptors start this first cyclin-depleted cycle in a dephosphorylated state), but subsequent Plk4 oscillations rapidly dampen as the individual Asl receptors lose synchrony, and the system tends toward a steady state—where some of the centriolar Asl receptors are Plk4-bound and being phosphorylated, while others are not Plk4-bound and are being dephosphorylated (Figure S8H). Intriguingly, the inherent noise in the system generated stochastic Plk4 oscillations that could plausibly drive centriole duplication (Figure S8H)—potentially mimicking the stochastic Plk4 oscillations and centriole duplication events that we observe in the cyclin-depleted embryos (Figure 5B).

In this model, each Asl receptor effectively behaves as an independent oscillator—alternating between a Plk4-bound form that is being phosphorylated and a non-Plk4-bound form that is being dephosphorylated. In the presence of the CCO, the Asl receptors generate coordinated Plk4 oscillations because the CCO synchronizes them every nuclear cycle by providing a coordinated burst of PPTase activity during mitosis.

### Plk4 Oscillations Are Detectable in Non-dividing Mouse Liver Cells and Can Be Entrained by the Circadian Clock

**Figure 7:**
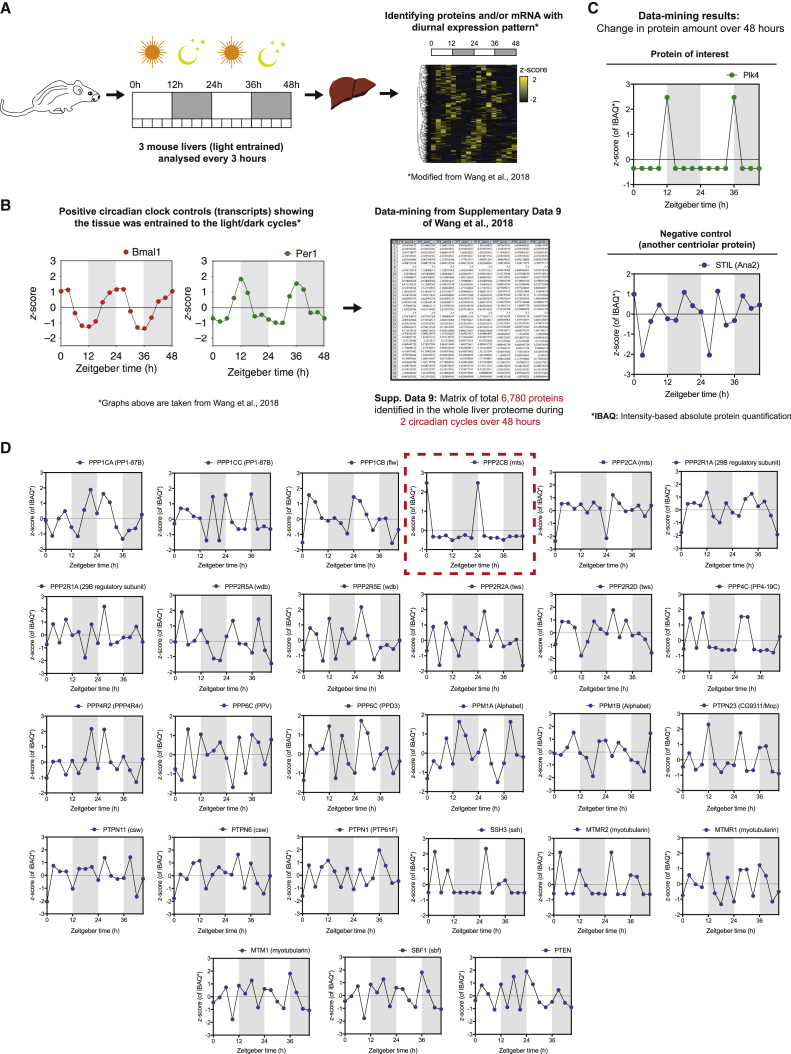
Plk4 Levels May Autonomously Oscillate in Mouse Liver Cells Entrained by the Circadian Clock Where Levels of PP2A Catalytic Subunit Oscillates Precisely out of Phase (A) Diagram shows the workflow used by Wang et al. (2018) to obtain a diurnal proteome of the whole liver of light/dark-entrained mice. (B) Graphs reproduced from Wang et al. (2018) show the relative diurnal expression of the circadian clock transcripts Bmal1 and Per1 as internal controls. We re-analyzed the diurnal proteome produced in this study—comprising a matrix of *Z* scores for 6,780 proteins identified during 2 circadian cycles (supplemental dataset 9 from Wang et al. [2018]). (C) Graphs we derived show the relative protein levels of Plk4 and the cartwheel component STIL (Ana2 in flies). Plk4 levels strongly spike in a periodic manner every circadian cycle, whereas STIL levels appear to randomly fluctuate and show neither a discernible pattern of oscillation nor any entrainment to the circadian clock. Because these cells are generally not proliferating, centrioles should not be duplicating, so the Plk4 oscillations are presumably sub-threshold for centriole biogenesis. Thus, Plk4 oscillations are detectable in non-dividing mammalian cells, where they are entrained by the circadian clock. (D) Graphs examine in the non-dividing liver cells the behavior of mouse homologs of all the mitotic PPTase subunits that function in flies (Chen et al., 2007). Among the 27 PPTase subunits examined, only PPP2CB (highlighted with a red dotted frame) exhibited a clear oscillatory behavior that is similar to Plk4, and the period of these oscillations was precisely out of phase with the Plk4 oscillation.

In species as distant as cyanobacteria and mammals, the CCO can be entrained to the circadian clock (Matsuo et al., 2003, Yang et al., 2010). We wondered, therefore, whether the autonomous Plk4 oscillation could also be entrained by the circadian clock. We examined a recently published diurnal proteome from non-regenerating mouse liver (Wang et al., 2018), where hepatocytes, the major building blocks of the liver, are largely quiescent (Friedman, 2000). Several key cell-cycle regulators (such as Cdk1, cyclin E, cyclin B1, and Plk1) were not detectable at any stage of the diurnal cycle, confirming that these cells were largely quiescent. In contrast, Plk4 protein (but not transcript) levels exhibited a striking oscillation that was entrained to the light/dark cycles (Figures 7A–7C). We presume that this oscillation is sub-threshold for centriole biogenesis—because centrioles should not be duplicating in these non-dividing cells—and simply reflects the ability of the Plk4 system to oscillate in a way that can be entrained by the circadian clock.

In our model, a mitotic PPTase that dephosphorylates Asl-receptors out of phase with Plk4 is required to generate Plk4 oscillations (Figures S4A and S4B). We therefore used the mouse dataset to examine the behavior of the mouse homologs of all the mitotic PPTase subunits that function in flies (Chen et al., 2007). Among the 27 PPTase subunits examined, only PPP2CB exhibited a clear oscillatory behavior that is similar to Plk4, and the period of these oscillations was precisely out of phase with the Plk4 oscillation (Figure 7D, highlighted with a red dotted frame). Intriguingly, PPP2CB is the homolog of Mts, the catalytic subunit of PP2A in *Drosophila* that localizes to centrosomes specifically during mitosis in fly cells, and its knockdown leads to centrosome duplication defects (Dobbelaere et al., 2008). Thus, PP2A is an excellent candidate for the PPTase that may normally dephosphorylate centriolar Asl during mitosis.

Remarkably, ∼8% of the ∼6,800 proteins in the mouse dataset exhibited a 24 h-entrained oscillatory behavior. It is unclear why so many proteins oscillate in this way, or whether any of these oscillations are of functional significance. Nevertheless, these observations indicate that there are many other proteins, and so perhaps many different biological processes, that have a largely under-appreciated ability to oscillate.

### Concluding Remarks

There is great interest in determining the physical and molecular principles that cells use to regulate the biogenesis of their organelles (Liu et al., 2018, Mukherji and O’Shea, 2014). The idea that an organelle-specific oscillation could time and execute organelle biogenesis has, to our knowledge, not been proposed previously. We suggest that the Plk4 centriole oscillation could be a paradigm for a general mechanism describing the regulation of organelle biogenesis: oscillations in the levels/activity of key regulatory factors essential for organelle biogenesis could precisely time the initiation and duration of the growth process, ensuring that organelles grow at the right time and to the appropriate size. In such a model, the CCO and circadian clocks could act simply as “phase-lockers” (Lu and Cross, 2010, Morgan, 2010), whose function is to entrain the phase of a network of autonomous oscillators to ensure that biological processes occur in a coordinated manner.

## STAR★Methods

### Key Resources Table

**Table:** 

REAGENT or RESOURCE	SOURCE	IDENTIFIER
**Antibodies**		

Mouse anti-GFP	Roche	RRID: AB_390913
Mouse anti-Actin	Sigma	RRID: AB_476730
HRPO-linked anti-mouse IgG	Sigma / GE Healthcare	Cat# GENA931

**Chemicals, Peptides, and Recombinant Proteins**		

QuikChange II XL mutagenesis kit	Agilent Technologies	Cat# 200521
Q5 Site Directed Mutagenesis kit	New England Biolabs	Cat# E0554S
Voltalef grade H10S oil	Arkema	N/A
Alexa Fluor 488 NHS Ester	Thermo Fisher Scientific	Cat# A20000

**Experimental Models: Organisms/Strains**		

*D. melanogaster*: Plk4-mNeonGreen	This paper	N/A
*D. melanogaster*: Plk4^Aa74^ (Plk4 null mutant)	Aydogan et al., 2018	FlyBase ID: FBab0049012
*D. melanogaster*: Asl-mKate2	This paper	N/A
*D. melanogaster*: Sas-6-mCherry	Rogers et al., 2008	N/A
*D. melanogaster*: CycB^2^	Jacobs et al., 1998	FlyBase ID: FBal0094855
*D. melanogaster*: grp^fsA4^	Sibon et al., 1997	FlyBase ID: FBal0062815
*D. melanogaster*: Asl-GFP	Blachon et al., 2008	FlyBase ID:FBtp0040947
*D. melanogaster*: asl^B46^	Baumbach et al., 2015	FlyBase ID: FBal0343439
*D. melanogaster*: Plk4-GFP	Aydogan et al., 2018	FlyBase ID: FBal0343977
*D. melanogaster*: Asl-mCherry	Conduit et al., 2015	FlyBase ID: FBal0343645
*D. melanogaster*: Sas-6-GFP	Aydogan et al., 2018	FlyBase ID: FBtp0131375
*D. melanogaster*: Asl-13A-mKate2	This paper	N/A
*D. melanogaster*: Asl-13A	This paper	N/A
*D. melanogaster*: Asl	This paper	N/A
*D. melanogaster*: wee1^∗^	(Homozygous viable mutant derived fromPrice et al., 2000; courtesy of Prof. Shelagh Campbell)	N/A
*D. melanogaster*: Plk4-mNeonGreen, Plk4^Aa74^ / Plk4-mNeonGreen, Plk4^Aa74^	This paper	N/A
*D. melanogaster*: Asl-mKate2 / Cyo; Plk4-mNeonGreen, Plk4^Aa74^ / Plk4-mNeonGreen, Plk4^Aa74^	This paper	N/A
*D. melanogaster*: Sas-6-mCherry / +; Plk4-mNeonGreen, Plk4^Aa74^ / Plk4-mNeonGreen, Plk4^Aa74^	This paper	N/A
*D. melanogaster*: CycB^2^ / +; Plk4-mNeonGreen, Plk4^Aa74^ / Plk4-mNeonGreen, Plk4^Aa74^	This paper	N/A
*D. melanogaster*: grp^fsA4^ / +; Plk4-mNeonGreen, Plk4^Aa74^ / Plk4-mNeonGreen, Plk4^Aa74^	This paper	N/A
*D. melanogaster*: Asl-GFP / Asl-GFP; asl^B46^ / asl^B46^	This paper	N/A
*D. melanogaster*: Oregon-R (Wild-type strain)	Kyoto Stock Center	FlyBase ID: FBst0324696
*D. melanogaster*: Asl-mKate2, asl^B46^ / +	This paper	N/A
*D. melanogaster*: Plk4-GFP / Cyo; Plk4^Aa74^ / Plk4^Aa74^	Aydogan et al., 2018	N/A
*D. melanogaster*: Plk4-mNeonGreen, Plk4^Aa74^ / Plk4^Aa74^	This paper	N/A
*D. melanogaster*: Asl-mCherry / +; Plk4^Aa74^ / +	This paper	N/A
*D. melanogaster*: Plk4-mNeonGreen / +; Plk4-mNeonGreen, Plk4^Aa74^ / Plk4^Aa74^	This paper	N/A
*D. melanogaster*: Plk4-mNeonGreen / +; Plk4-mNeonGreen, Plk4^Aa74^ / asl^B46^, Plk4^Aa74^	This paper	N/A
*D. melanogaster*: Asl-GFP / +; asl^B46^ / asl^B46^	This paper	N/A
*D. melanogaster*: Plk4-GFP / Cyo; asl^B46^, Plk4^Aa74^ / Plk4^Aa74^	This paper	N/A
*D. melanogaster*: Sas-6-GFP / +; asl^B46^ / +	This paper	N/A
*D. melanogaster*: Asl-13A-mKate2 / Asl-13A-mKate2; asl^B46^ / asl^B46^	This paper	N/A
*D. melanogaster*: Asl-mKate2 / Asl-mKate2; asl^B46^ / asl^B46^	This paper	N/A
*D. melanogaster*: Asl-13A / +; Plk4-mNeonGreen, Plk4^Aa74^ / Plk4-mNeonGreen, Plk4^Aa74^	This paper	N/A
*D. melanogaster*: Asl / +; Plk4-mNeonGreen, Plk4^Aa74^ / Plk4-mNeonGreen, Plk4^Aa74^	This paper	N/A
*D. melanogaster*: wee1^∗^ / wee1^∗^; Plk4-mNeonGreen, Plk4^Aa74^ / Plk4-mNeonGreen, Plk4^Aa74^	This paper	N/A

**Oligonucleotides**		

Primers to introduce the NheI restriction enzyme sites into the mCherry C-terminal Gateway vector, see Table S1.	Invitrogen, Thermo Fisher Scientific	N/A
Primers to replace the mCherry tag with mNeonGreen by homologous recombination on the destination vector, see Table S1.	Invitrogen, Thermo Fisher Scientific	N/A
Primers to replace the mCherry tag with mKate2 by homologous recombination on the destination vector, see Table S1.	Invitrogen, Thermo Fisher Scientific	N/A
Primers to remove the NheI restriction enzyme sites from the destination vector via site-directed mutagenesis (mNeonGreen vector), see Table S1.	Invitrogen, Thermo Fisher Scientific	N/A
Primers to remove the NheI restriction enzyme sites from the destination vector via site-directed mutagenesis (mKate2 vector), see Table S1.	Invitrogen, Thermo Fisher Scientific	N/A
Primers to amplify Cyclin A, B or B3, see Table S1.	Invitrogen, Thermo Fisher Scientific	N/A
Primers to introduce various site directed mutations for Asl-13A construct, see Table S1.	Invitrogen, Thermo Fisher Scientific	N/A
Primers to delete mKate2 to generate endogenous Asl-13A construct without a fluorescent tag, see Table S1.	Invitrogen, Thermo Fisher Scientific	N/A
Primers to generate endogenous Asl construct without a fluorescent tag, see Table S1.	Invitrogen, Thermo Fisher Scientific	N/A

**Recombinant DNA**		

mCherry C-terminal Gateway vector	Basto et al., 2008	N/A
pDONR-Zeo vector	Thermo Fisher Scientific	Cat# 12535035
mNeonGreen vector	Shaner et al., 2013	N/A
mKate2 vector	Shcherbo et al., 2009	N/A
Asl-mKate2 P-element transformation vector	This study	N/A

**Software and Algorithms**		

Fiji (ImageJ)	National Institutes of Health	https://imagej.nih.gov/ij/
TrackMate	Tinevez et al., 2017	https://imagej.net/TrackMate
Prism 7 and 8	GraphPad	https://www.graphpad.com/scientific-software/prism/
Scipy’s *find_peaks* function	Jones et al., 2001	https://docs.scipy.org/doc/scipy/reference/generated/scipy.signal.find_peaks.html
Asymmetric baseline smoothing	Eilers and Boelens, 2005	N/A
Zen Black Software	Zeiss	https://www.zeiss.com/microscopy/us/products/microscope-software/zen.html
FoCuS-Point Software	Waithe et al., 2016	N/A
The equations used for mathematical modeling and regressions	This paper	https://github.com/RaffLab/centriole_oscillator_model
Python script to automate PeCoS analysis	This paper	https://github.com/RaffLab/centriole_oscillator_model

### Resource Availability

#### Lead Contact

Further information and requests for resources and reagents should be directed to and will be fulfilled by the Lead Contact, Jordan W. Raff (jordan.raff@path.ox.ac.uk).

#### Materials Availability

All unique/stable reagents generated in this study are available from the Lead Contact without restriction, unless for commercial application, in which case a completed Materials Transfer Agreement will be requested. There is restriction to the availability of dsRNA cocktails produced in this study, as they only last for ∼6 months without degradation (if preserved at conditions indicated in the STAR Methods), and therefore these cocktails are recommended to be made fresh using the protocol described in the STAR Methods. Fly alleles and plasmids (with original source species) generated in this study will be requested by FlyBase administration to deposit onto FlyBase public archives within 6 months following the publication of this study. Compound and recombinant flies are deposited to the Lead Contact’s laboratory stocks (without direct public access), but are available without restriction upon request.

#### Data and Code Availability

The codes generated to perform mathematical modeling and regressions are available in the following web link: < https://github.com/RaffLab/centriole_oscillator_model >. The code generated to automate PeCoS analysis procedure is available in the following web link: < https://github.com/RaffLab/PeCoS >. Source 3D time-lapse spinning-disk confocal micrographs and SIM reconstruction datasets supporting the current study are of sizes between 10 and 20GB for each experiment (exceeding the current upload limits of public repositories) and therefore have been deposited in Open Microscopy Environment (OMERO) repository. These are available without restriction, via file transfer systems, when requested from the Lead Contact – unless for commercial application, in which case a completed Materials Transfer Agreement will be requested.

### Experimental Model and Subject Details

#### *D. melanogaster* stocks and husbandry

The specific *D. melanogaster* stocks used, generated and/or tested in this study are listed in Key Resources Table. To generate Plk4-mNeonGreen and Asl-mKate2 constructs: 1) NheI restriction enzyme sites were introduced into an mCherry C-terminal Gateway vector (Basto et al., 2008), using the Quikchange II XL mutagenesis kit (Agilent Technologies). 2) The mCherry tag was replaced with either mNeonGreen (Shaner et al., 2013) (Allele Biotechnology) or mKate2 (Shcherbo et al., 2009) tags by homologous recombination via In-fusion Cloning (TaKaRa). 3) NheI restriction enzyme sites were removed via site-directed mutagenesis, using the Quikchange II XL mutagenesis kit (Agilent Technologies). These vectors were recombined via Gateway technology to pDONR-Zeo vectors (Thermo Fisher Scientific) where the genetic regions of either *Plk4* (Aydogan et al., 2018) or *asl* (Novak et al., 2014) were previously cloned from 2 kb upstream of the start codon up to (but excluding) the stop codon. Similary, to generate an Asl construct without any fluorescent tag, the endogeneous Asl stop codon was introduced to the Asl pDONR by site-directed mutagenesis, using the Quikchange II XL mutagenesis kit.

To generate the Asl-13A-mKate2 construct, 13 point mutations (Figure S4C) were introduced into the endogenous Asl-mKate2 P-element transformation vector (this study) through NEB Q5 Site Directed Mutagenesis (NEB #E0554S) in four sequential mutagenesis steps. To generate the endogenous Asl-13A construct without any fluorescent tag, the mKate2 coding sequence was removed, and a stop codon was introduced immediately following the Asl coding sequence in the eAsl-13A-mKate2 vector (described above) using NEB Q5 Site Directed Mutagenesis.

Primer sequences used to generate these constructs are listed in Table S1. Transgenic lines were generated using standard P-element mediated transformation by the Fly Facility in the Department of Genetics, University of Cambridge (Cambridge, England, UK) or BestGene Inc. (USA). Flies were maintained at 18°C or 25°C on *Drosophila* culture medium (0.77% agar, 6.9% maize, 0.8% soya, 1.4% yeast, 6.9% malt, 1.9% molasses, 0.5% propionic acid, 0.03% ortho-phosphoric acid, and 0.3% nipagin) in vials or bottles.

### Method Details

#### Hatching experiments

To measure embryo hatching rates, 0-3 h embryos were collected and aged for 24 h, and the % of embryos that hatched out of their chorion was calculated.

#### Synthesis of double-stranded RNA

Double-stranded RNAs (dsRNAs) against cyclins A, B and B3 were synthesized essentially as described previously (McCleland and O’Farrell, 2008). Primer sequences used for gene amplification are listed in Table S1. The resulting RNA was precipitated with 8 μL of 3M Na-Acetate and 220 μL of 100% ethanol before washing with 70% cold ethanol. The RNA pellets were air-dried and resuspended in 30 μL of RNase-free diethylpyrocarbonate-treated water (Thermo Fisher Scientific). To generate double-stranded molecules, RNAs were placed in a 67.5°C water bath for 30 min, and allowed to cool to room temperature over 90 min. Unincorporated UTPs were removed using CHROMA SPIN-100-DEPC-H_2_O columns (Clontech) according to the manufacturer’s instructions. To confirm the synthesis of the correct RNA product, 3 μL of the final reaction was subjected to electrophoresis on a 1.5% agarose gel using 2xRNA loading buffer (Thermo Fisher Scientific). A 1:1 mix of RNA and loading buffer was heated to 65°C for 5 min and then placed on ice to denature any secondary structure of RNA.

#### Embryo collections and dsRNA injections

For embryo collections, 25% cranberry-raspberry juice plates (2% sucrose and 1.8% agar with a drop of yeast suspension) were used. Embryos for imaging experiments were collected for 1h at 25°C, and aged at 25°C for ∼45–60 min. Embryos were dechorionated by hand, mounted on a strip of glue on a 35-mm glass-bottom Petri dish with 14 mm micro-well (MatTek), and were left to desiccate for 1 min at 25°C. After desiccation, the embryos were covered with Voltalef grade H10S oil (Arkema). Embryos for dsRNA injection experiments were treated in the same way except that the desiccation period was increased to 5-6 min. Embryos were injected with dsRNA at a needle concentration of 0.6–0.8 mg/ml.

#### Immunoblotting

Immunoblotting was performed as described previously (Aydogan et al., 2018). Primary antibodies used in this study are as follows: mouse anti-GFP (Roche; RRID: AB_390913) and mouse anti-Actin (Sigma; RRID: AB_476730). Both the antibodies were used at 1:500 dilution in blocking solution (Aydogan et al., 2018). For all blots, 10, 20 or 30 staged early embryos were boiled in sample buffer and loaded in each lane. The incubation period for primary antibodies was 1 h (or overnight at 4°C). Membranes were quickly washed 3x in TBST (TBS and 0.1% Tween 20) and then incubated with HRPO-linked anti-mouse IgG (both GE Healthcare) diluted 1:3,000 in blocking solution for 45 min. Membranes were washed 3x15min in TBST and then incubated in SuperSignal West Femto Maximum Sensitivity Substrate (Thermo Fisher Scientific). Membranes were exposed to film using exposure times that ranged from < 1 to 60s.

#### Image acquisition, processing, and analysis

##### Spinning disk confocal microscopy

Living embryos were imaged at room temperature using a system equipped with an EM-CCD Andor iXon+ camera on a Nikon Eclipse TE200-E microscope using a Plan-Apochromat 60x/1.42-NA oil DIC lens, controlled with Andor IQ2 software. Confocal sections of 17 slices at 0.5μm intervals were collected every 30 s. A 488nm laser was used to excite mNeonGreen and GFP, and a 568nm laser was used to excite mCherry and mKate2. Emission discrimination filters were applied when mNeonGreen and mCherry were imaged together.

Post-acquisition image processing was performed using Fiji (National Institutes of Health). Maximum-intensity projections of the images were first bleach-corrected with Fiji’s *exponential fit* algorithm, and background was subtracted using the *subtract background* tool with a rolling ball radius of 10 pixels. Plk4-NG, Sas-6-mCherry or -GFP, and Asl-mCherry or -GFP were tracked using the Fiji plug-in TrackMate (Tinevez et al., 2017) with a track spot diameter size of 1.1 μm. When Plk4-NG was continuously monitored over cycles 11-13, the maximum intensity projections were limited to ± 5 slices from the central plane of the nuclei, as the nuclei and centrosomes progressively get closer to the embryo cortex at successive cycles. This processing more accurately compares the dynamics of centriolar Plk4-NG at successive cycles by avoiding fluctuations due to the varying depths of the centrioles in the embryo. The regressions for the centriole growth curves (Sas-6-GFP or -mCherry) were calculated in Prism 7 (GraphPad Software), as described previously (Aydogan et al., 2018). The regressions for the Plk4 oscillation curves (Plk4-NG) were calculated using the *nonlinear regression* (curve fit) function in Prism 7. Discrete Plk4 oscillation curves in S-phase were initially fitted against four different functions to assess the most suitable regression model: 1) Lorentzian, 2) Gaussian, 3) Increase – Constant – Decrease, and 4) Increase – Decrease. Among these models, Lorentzian best fit the data (Figure S1D), so all the discrete Plk4 oscillation curves in S-phase were regressed using this function. The Lorentzian and Gaussian functions are described in Prism 7, while the latter two functions are in-house algorithms (Alvarez-Rodrigo et al., 2019).

In order to plot the dynamics of Plk4-NG and Sas-6-mCherry together (Figures 2, S3A, and S3B), the highest mean fluorescence signal for each tag was normalized to 1 and was accordingly scaled across cycles 11-13 (the scaling factor for Plk4-NG was calculated from the data shown in Figures 1B and 1C). Note that the amplitude of the Plk4 oscillation does not appear to decrease significantly between nuclear cycles 11-13 in the data shown in Figure 2A—in contrast to the Plk4 oscillations shown in Figure 1B. This is not because of the scaling procedure applied to the data shown in Figure 2A (described above), but rather because embryos that failed to grow their centrioles were excluded from the analysis shown in Figure 2A. The amplitude of the Plk4 oscillations was lower in these embryos (Figure 2C), so embryos with low amplitude Plk4 oscillations were effectively excluded from the analysis shown in Figure 2A. Almost all of these excluded embryos were at nuclear cycle 13, so the “average” oscillation at nuclear cycle 13 is in reality an average of only those embryos that had a relatively high amplitude Plk4 oscillation.

In all the imaging experiments, the beginning of S-phase was taken as the time at which the old and new mother centrioles were first detected to separate from each other (termed “centrosome separation” or “CS”). Entry into mitosis was taken as the time of nuclear envelope breakdown (NEB), which could be determined in our movies by adjusting the contrast to visualize when the cytosolic pool of the fluorescent protein was first observed to enter into the nucleus.

#### Analysis of centriole “fertility” in embryos injected with dsRNA against cyclin A-B-B3

In experiments where we depleted embryos of mitotic cyclins during early rounds of nuclear division, we observed qualitatively that “fertile” centrioles exhibited distinct Plk4-NG fluorescence peaks that often appeared to correlate with centriole duplication events, while “sterile” centrioles exhibited no obvious peaks (Figure 5B). To test if we could more quantitatively distinguish between fertile and sterile centrioles, we computationally analyzed all 81 centrioles that we could track throughout the observation period in 3 different embryos. We first assessed the average signal-to-noise ratio (SNR) of Plk4-NG fluorescence of each centriole over the entire observation period and found that fertile centrioles exhibited a significantly higher SNR than sterile centrioles—assessed using a t test assuming equal variance (Figure S8B). The distribution of SNR within sterile and fertile centriole signals was unimodal and symmetrically distributed (Figure S8C), so we attempted to classify centrioles in an unbiased way by thresholding the SNR. Based on the bimodality of the SNR, an automatic threshold was determined from the data using Otsu thresholding (*red dashed* line Figure S8C); the classification performance was summarized in a visual confusion matrix, which shows the proportion of correctly and falsely classified signals (Figure S8D). This unbiased computational method successfully classified ∼74% of the fertile centrioles and ∼71% of the sterile centrioles.

##### Peak Calling

We next tested whether computationally identified peaks in the Plk4-NG signal were correlated with centriole duplication events. Plk4-NG peaks were only called on signals whose fluctuation (as measured by signal-to-noise ratio, SNR) was greater than a certain defined threshold (0.1, see below). A peak was defined only as a local maximum in intensity. To call a peak, the Plk4-NG signal intensity was compared to the signal intensity at neighboring times. Here an unbiased distance = 1 was set, that is, an intensity at time t is a peak only if the intensity is higher than those at both t−1 and t+1. To filter noise detections, a threshold of 0.1 was placed on the peak prominence. Peak prominence measures the extent to which a detected peak stands out from its surrounding – it is defined as the vertical distance between the peak and its lowest contour line (Scipy’s *find_peaks* function) (Jones et al., 2001). The choice of 0.1 as a threshold was guided by comparing a peaks predictive power given no cut-off with the (ground truth) duplication time. This analysis indicated that the optimal peak prominence cut-off—i.e., the point at which the power of the peaks to predict duplication events (see below) sharply drops off—was 0.12 (*green dot*, Figure 5E). The observed steep drop-off in predictive power below this threshold supports the view that that there is likely to be a minimal amount of centriolar Plk4-NG that is necessary to trigger duplication under these conditions. Moreover, this unbiased computational approach identified ∼4x as many peaks in the fertile centrioles when compared to the sterile centrioles.

To determine whether the filtered Plk4-NG peaks were predictive of centriole duplication, we determined all the peaks above the 0.1 threshold for the fertile centriole signals and assessed whether these peaks could be used to “retrieve” the real or relevant time points for centriole duplication. The performance of such retrieval can be evaluated using “precision” (the number of relevant retrievals among all retrieved instances—in this case the number of Plk4 peaks associated with a centriole duplication event divided by the total number of Plk4 peaks) and “recall” (the number of relevant instances retrieved of the total relevant instances—in this case the number of Plk4 peaks associated with a centriole duplication event divided by the total number of centriole duplication events), as defined below.

$$Precision\;=\;\frac{Number\;of\left(Relevant\;\&\;Retrieved\right)}{Number\;of\;\left(Retrieved\right)},\;$$

$$Recall\;=\frac{Number\;of\left(Relevant\;\&\;Retrieved\right)}{Number\;of\left(Relevant\right)}$$

The evaluation of such a system naturally depends on the cut-off to call a positive match between the Plk4-NG signal peak and its corresponding centriole duplication time. Too small a cut-off (e.g., 0 minutes) is unrealistic: no system can predict time perfectly; while too large a cut-off (e.g., 15 min) is too lenient and non-specific. Figure 6C plots the precision evaluated over all centrioles for different temporal cut-offs attempting to uniquely match Plk4-NG peaks to the nearest duplication time within a given time window. The elbow point (*red dashed* line) at 5 min was selected as an appropriate cut-off with a precision of ∼80%. (Note that the recall was not plotted in this graph, but it exhibits a similar behavior to the precision: the *Number of (Relevant)* = 52, while the *Number of (Retrieved)* = 49). This temporal cut-off can also be interpreted as an estimate of the temporal accuracy to which Plk4-NG peak time associates with centriole duplication time. For comparison, we also derived the mean temporal separation distance of peaks and duplication events, if the same number of experimental centriole duplication times were randomly distributed over the same time interval for each embryo. 1000 simulations were run per embryo to produce a distribution. Across all embryos, an average temporal separation distance (for randomly distributed duplication times) was 10.5 minutes (data not shown), twice as long as the chosen 5 min cut-off, thus the association is not coincidental.

In addition, we assessed the precision and recall performance over different possible threshold values (based on peak prominence) used to call a Plk4 ‘peak’. To do this, we computed the precision-recall curve. All detected Plk4 peaks (*Black* dots; Figure 5E) were ranked according to their peak prominence from high to low and were assigned uniquely to a duplication event according to a 5 min time window for determining a positive match. Peaks that could not be uniquely assigned in such a manner were regarded as ‘negatives’. The graph then plots the precision, recall values if the threshold for calling a peak were set as the peak prominence value of each peak in descending order. Beyond the detected peak associated with a peak prominence of 0.12 (i.e., points right of this point), the precision drops sharply. At this threshold, precision and recall are jointly optimized. This suggests that a minimum level of Plk4-NG peak fluorescence intensity is required to predict duplication. The ability of Plk4 peaks to predict duplication across all peak prominences (over the selected time window of 5 min) is quantified by the integrated area under the curve or average precision (AP_5min_). If there were no overall correlation between a Plk4 peak and a duplication event, AP_5min_ would be 51.5% (given by # duplications / (# duplications + # peaks)); the score of ∼75% indicates a strong overall correlation (Figure 5E).

Finally, the correlation between the Plk4-NG peaks and times of centriole duplication was examined (Figure 5F), which provided an alternative accuracy test. Plk4-NG peaks were uniquely matched to the nearest centriole division times without using a temporal cut-off over individual centrioles from three independent embryos. Pearson correlation r, *R*^*2*^ and *P value*s are reported as goodness of fit. The fitted regression line, y=0.87x+3.69. Together, these unbiased computational analyses indicate that the Plk4 oscillations at individual centrioles are highly correlated with the time at which these centrioles duplicate.

#### Spatiotemporal heatmap of centriole duplications

To visually assess whether there is bias as to where and when centriole duplications happen, the (x,y) position of all duplicating centrioles were overlaid on the (x,y) positions of all centrioles (duplicating and non-duplicating at all times; *black* dots) (Figure S8E). To grade the temporal sequence of centriole separations, duplication points were colored blue to red. To enable comparison across embryos relative to the same geometric reference, the embryonic width and anterior-posterior were set as *x*- and *y*-axes, respectively, by applying a principal component analysis on the extracted (x,y) positions of all centrioles.

#### Spatial clustering assessment of centriole duplications

To statistically measure whether centriole duplications are enriched at particular spatial regions over time, Ripley *K* statistics was used (Ripley, 1976). In the field of spatial statistics, given a set of (x,y) points, the Ripley *K* statistic detects deviations from spatial homogeneity at different distances between points or spatial scales (e.g., such analyses are heavily used in geophysics to map out the spatial distribution of natural disasters and in crime statistics for detecting high incidence areas). For a dataset of n points, the Ripley *K* statistic, $K^{Ripley}\left(d\right)\;$is the mean spatial occurrence of two points, point i and j having a separation distance, $d_{ij}$ less than the search distance threshold of d:

$$K^{Ripley}\left(d\right)=\frac{1}{\lambda }\sum_{i\neq j}\frac{I\left(d_{ij}<d\right)}{n}$$

λ is the average density of points (estimated as n/A where the number of total points, n is divided by the area of the region containing all points, *A*), and I is the indicator counting function (I = 1 if its operand is true, 0 otherwise). Thus, if points are homogeneously spread in 2D, the Ripley *K* statistic should vary quadratically as $\pi d^{2}$. The basic test assumes (x,y) points occur at any spatial position continuously in the image. However, centrioles only duplicate in certain discrete positions within fly embryos. Thus, to examine evidence of spatial clustering from the natural distribution of centrioles, we assessed difference in the Ripley *K* statistics computed from the (x,y) positions of all duplicating centrioles and the (x,y) positions of all centrioles accumulated over time.

#### Plk4-NG smooth curve fitting and parameter extraction

To enable accurate extraction of signal parameters from the Plk4-NG oscillations, y(t) the signals were robustly fit to a smooth 1D function. Here a mixture of N Gaussian and linear trend was used with the following functional form to enable local modeling of the peak and troughs of signals:

$$y\left(t\right)=\left(A+Bt\right)+\sum_{i=1}^{N}C_{i}e^{-\frac{{\left(t-\mu_{i}\right)}^{2}}{\sigma_{i}^{2}}}$$

where t is time, A,B are the constant and slope of a linear trend line, and $C_{i},\;\mu_{i},\;\sigma_{i}$ are the amplitude, time and temporal duration of the i^th^ Gaussian, respectively. This function was fit in two steps. In the first step A,B was fit by applying least-squares linear regression on the baseline trend line that is extracted from asymmetric baseline smoothing (Eilers and Boelens, 2005). In the second step, peak and trough positions were first detected on the de-trended signal, y'(t) after subtraction of the fitted trend line in the first step from the original signal, y(t), so as to determine the number N of Gaussians to fit. The mixture of Gaussians was then fit by iterative non-linear regression using a robust Cauchy loss function. From the fit signal, $y{\left(t\right)}_{fitted}$, peak and trough positions were re-detected, and the following signal parameters were extracted (Figure 6):

• **Acceleration rate** (ΔFluo.A.U./secs)**:** The maximum rate of increase in fluorescence between successive time points during a trough to peak oscillation phase.
• **Deceleration rate** (ΔFluo.A.U./secs)**:** The maximum rate of decrease in fluorescence between successive time points during a peak to trough oscillation phase.
• **Oscillation peak time** (s)**:** The time point corresponding to the maximum fluorescence.
• **Oscillation trough time** (s) **into mitosis:** The end point of a trough after which the fluorescence begins to accelerate upward.

The method described here was also used to determine the period of Plk4 oscillations (measuring peak-to-peak time; see Results and Discussion) both in normal embryos and in embryos where dsRNA was injected against cyclins A, B and B3 to halt the progression of cell cycle.

#### 3D-Structured Illumination Microscopy (3D-SIM)

Living embryos were imaged at room temperature using a DeltaVision OMX V3 Blaze microscope (GE Healthcare). The system was equipped with a 60x/1.42-NA oil UPlanSApo objective (Olympus Corp.), 488nm and 593nm diode lasers, and Edge 5.5 sCMOS cameras (PCO). Spherical aberration was reduced by matching the refractive index of the immersion oil (1.514) to that of the embryos. 3D-SIM image stacks consisting of six slices at 0.125μm intervals were acquired in five phases and from three angles per slice. The raw acquisition was reconstructed using softWoRx 6.1 (GE Healthcare) with a Wiener filter setting of 0.006 and channel-specific optical transfer functions (OTFs). Filters used for the green and red channels were a 540/80 center band pass filter and a 605-long pass filter, respectively. For two-color 3D-SIM, images from green and red channels were registered with the alignment coordination information obtained from the calibrations using 0.2μm-diameter TetraSpeck beads (Thermo Fisher Scientific) in the OMX Editor software. The SIMCheck plug-in in ImageJ (National Institutes of Health) was used to assess the quality of the SIM reconstructions (Ball et al., 2015); only images that passed this test were used.

#### Mathematical modeling and its experimental validation

##### Model 1: A simple mathematical model of discrete S-phase Plk4 oscillations

Figures 3A and 3B, specify a regulatory network where Plk4 binds to an Asl receptor with high affinity; this activates Plk4, allowing it to phosphorylate itself and Asl multiple times. After a certain number of phosphorylations, Asl switches to a new state that binds Plk4 with low affinity. As a result, Plk4 unbinds, leaving Asl in a phosphorylated, low affinity state. We suspect that Asl is normally dephosphorylated by a phosphatase in M-phase, which “resets” it to a high-affinity state in preparation for the next oscillation in S-phase (this additional step is considered in Model 2 below). In this first model, the gradual conversion of centriolar Asl to a low affinity binding state forms a time-delayed negative feedback loop wherein Asl effectively activates Plk4 to gradually promote its own inhibition. After making assumptions about the chemical kinetics of the system and imposing suitable initial conditions, the behavior of this regulatory network can be simulated by mapping it onto a set of coupled ordinary differential equations.

In the model, it is assumed that the diffusion of Plk4 is sufficiently fast such that it remains well-mixed in the cytoplasm and that centrioles are large macromolecular structures; this implies that Plk4 and Asl receptors on the centriole follow mass-action kinetics. Let [P] and $\left[A_{0}\right]$ denote the concentrations of unbound cytosolic Plk4 and unbound centriolar Asl, (per unit volume and area, respectively). Plk4 binds to Asl with the fixed rate constant k, and the rate constant of the reverse reaction is sufficiently small that any unbinding is ignored. Once Plk4 is bound to Asl, it can only unbind after it has phosphorylated Asl a certain number of times (*N)*. We denote by $\left[A_{0}^{*}\right]\;$ the concentration of Asl receptor that has bound Plk4, but has not yet been phosphorylated, and by $\left[A_{i}^{*}\right]$ the concentration of Asl receptors that have been phosphorylated *i* times. Throughout this paper, we use an asterisk superscript to denote an Asl receptor that has bound Plk4 and a numerical subscript to denote the phosphorylation state of the receptor. Each phosphorylation of Asl by Asl-bound Plk4 has rate constant $k_{2}$ and, following *N* phosphorylations, the Asl is switched to a state that binds Plk4 with very low affinity $\left[A_{N}^{*}\right]$. Once Asl has been converted to this low affinity state, Plk4 unbinds at rate $k_{3}$. The rate constant of the reaction where Plk4 binds to Asl receptor in this low affinity state is assumed to be sufficiently small that this reaction is ignored in the model.

Intuitively, k scales the affinity with which Plk4 binds to an Asl receptor. By mass-action kinetics, the rate of this reaction is given by $k\left[P\right]\left[A_{0}\right]$. It is assumed in the model that Plk4 is abundant enough in the cytoplasm that its concentration does not decrease over the few minutes of a single S-phase cycle; this assumption means that [P] remains constant over that time (see below for further discussion of this assumption). Therefore, the number of parameters in the model is reduced by introducing a new rate constant $k_{1}=k\left[P\right]$.

Using the assumptions above, the regulatory network in Figures 3A and 3B is simulated using the following set of ordinary differential equations which are solved over the time domain 0 ≤ *t* ≤ *S*, where *S* is the length of S-phase:

(1) $$\frac{d\left[A_{0}^{*}\right]}{dt}=k_{1}\left[A_{0}\right]-k_{2}\left[A_{0}^{*}\right]$$

(2) $$\frac{d\left[A_{1}^{*}\right]}{dt}=k_{2}\left[A_{0}^{*}\right]-k_{2}\left[A_{1}^{*}\right]$$

(3) $$\frac{d\left[A_{N}^{*}\right]}{dt}=k_{2}\left[A_{N-1}^{*}\right]-k_{3}\left[A_{N}^{*}\right]$$

(4) $$\frac{d\left[A_{0}\right]}{dt}=-k_{1}\left[A_{0}\right]$$

Appropriate initial conditions at t = 0 are,

(5) $$\left[A_{0}^{*}\right]={\hat{A}}_{0}^{*};\;\;\left[A_{0}\right]={\hat{A}}_{0};\;\;\;\left[A_{1}^{*}\right]=\left[A_{2}^{*}\right]=\cdots =\left[A_{N}^{*}\right]=0.$$

In Equation 5, the positive constant ${\hat{A}}_{0}^{*}$ is the initial amount of Plk4-bound Asl at the centriole at the start of each S-phase, which is determined experimentally for each cell cycle using the techniques described in the *Image acquisition, processing and analysis* section of STAR Methods. The constant $\hat{A}$ is the initial amount of unbound Asl, so the total amount of Asl in the system is given by $A_{tot\;}={\hat{A}}_{0}^{*}+\;{\hat{A}}_{0}$.

Since the model specified by (1), (2), (3), (4), (5) is a system of linear differential equations with constant coefficients, it has an analytical solution that can be expressed as a sum of exponentials. Values for the parameters ${\hat{A}}_{0}$, $k_{1}$, $k_{2}$, and $k_{3}$ can then be determined by fitting the curve $\left[A_{0}^{*}\right]\left(t\right)+\left[A_{1}^{*}\right]\left(t\right)+\cdots +\left[A_{N}^{*}\right]\left(t\right)$ to the experimentally measured data for the amount of Asl-bound Plk4 (i.e., the Plk4 that is recruited to the centriole) over time. Fitting was done using a *trust-region* algorithm to optimize a nonlinear least-squares penalty function.

##### Parameter fitting

The fitting was constrained to enforce that all parameters were positive, and $k_{1}$ and $k_{2}$ were taken to be less than 1. Each cycle was fitted individually using the discrete Plk4-NG oscillation data from S-phase of cycles 11, 12 and 13 (Figure 3C). Parameter values are shown in Data S1 (First, second and third charts). As explained below, the solutions to this model are very insensitive to variations in $k_{3}$ (see Data S1, the *Monte Carlo* analysis), so in the solutions presented here $k_{3}$ was kept at a constant value of 0.06906, which was the best-fit parameter value for cycle 12 (Data S1; first, second and third charts).

##### Picking the value of N, the number of phosphorylation sites:

In the model, we assumed that Asl had to be phosphorylated by Plk4 *N* times before it switched to a low-affinity state—indicated by variables $\left[A_{0}^{*}\right],\cdots ,\;\left[A_{N}^{*}\right]$. We tested the effect of the number of phosphorylation sites on the model solution by using N = 1, 4, 9, 14, or 16. The best fit curves for $\left[A_{0}^{*}\right]\left(t\right)+\left[A_{1}^{*}\right]\left(t\right)+\cdots +\left[A_{N}^{*}\right]\left(t\right)$ suggested that the model is a good fit for the data for any value of N > 4 (N = 1 (*R*^*2*^ = 0.9152), N = 4 (*R*^*2*^ = 0.9886), N = 9 (*R*^*2*^ = 0.9996), N = 14 (*R*^*2*^ = 0.9962) or N = 16 (*R*^*2*^ = 0.9931)). So, we use N = 9 corresponding to 10 phosphorylation sites of Asl (1 unbound, 9 bound with various stages of phosphorylation) in all subsequent modeling, although we note that any value above 4 works essentially equally well.

Data S1, first chart, shows that the *trust-region* algorithm finds a very good fit (*R*^*2*^ > 0.99) for the model to the experimental data (Figure 3C), but this provides little information about uniqueness of the fit as there may be other subsets of the parameter space that also provide a good fit to the data. To see if any such regions could be detected, the parameter space was further explored by using a *Metropolis-Hastings Markov chain Monte Carlo* algorithm. Four Markov chains were started at the positions in the parameter space specified in Data S1 (fourth chart). The *Monte Carlo* analysis in Data S1 shows the six two-dimensional traces of the four-dimensional parameter space. For clarity, only points that provided a good fit to the cycle 12 data (*R*^*2*^ > 0.95) are shown.

The results in Data S1 (the *Monte Carlo* analysis) reveal how sensitive the model is to changes in each parameter value. The model only fit the data well for a relatively narrow range of values for $k_{1}$, $k_{2}$, and ${\hat{A}}_{0}$. In contrast, the fits are mostly insensitive to $k_{3}$. This is likely, because the rate of phosphorylating Asl at multiple sites is relatively slow compared to the rate at which Plk4 is subsequently released from the multiply phosphorylated Asl—so the rate of release is not limiting. The Monte Carlo simulations also reveal correlations between $k_{1}$ and $k_{2}$, $k_{1}$ and ${\hat{A}}_{0}$, and $k_{2}$ and ${\hat{A}}_{0}$. For example, these results show that if ${\hat{A}}_{0}$ (the initial amount of unbound Asl receptor at the start of S-phase) is reduced, the model can still fit the data well if $k_{2}$ is decreased and $k_{1}$ is increased. While these results suggest that there is a single, continuous region of the parameter space that provides a good fit to the data, it is still possible that there are other such regions that the Markov chains in Data S1 (the *Monte Carlo* analysis) did not explore. However, the results in Data S1 (the *Monte Carlo* analysis, panel B) show that the points, which are identified at the center of the parameter region, provide the best fit to the data. This suggests the nonlinear least-squares minima found by the *trust-region* fitting is insensitive to the initial seed.

Interestingly, the best-fit parameters for cycles 11–13 showed that the biggest difference between the parameters of the Plk4 oscillations at each cycle is in *k*_1_—the rate at which Plk4 binds to Asl (which is dependent on the cytosolic concentration of Plk4). Although our model assumes that the cytosolic concentration of Plk4 remains constant during the S-phase period within each cycle, if the phosphorylated Plk4 molecules that are released from the Asl receptor are ultimately degraded—and there is good evidence that Asl activates Plk4 to promote Plk4 degradation (Klebba et al., 2015)—there could be a wave of phosphorylated-Plk4 degradation in the cytoplasm toward the end of S-phase. If so, the cytosolic levels of Plk4 would get successively lower at the start of each successive cycle, as our PeCoS analysis indicates is the case (Figure 3E).

The effects of reducing the genetic dose of Plk4 by half (*Plk4-NG*^*1/2*^ embryos—see Results and Discussion for details) were analyzed. Our PeCoS analysis indicated that there was a ∼45% drop in the cytosolic levels of the Plk4-NG protein in the *Plk4-NG*^*1/2*^ embryos (Figure S6C). When the model was fit to *Plk4-NG*^*1/2*^ oscillation, the best-fit (*R*^*2*^ = 0.996) parameter had a *k*_1_ value that was ∼39% of the control value (Data S1, second chart), so in reasonable agreement with the 45% drop in cytosolic Plk4 levels we measured experimentally. These parameter values also suggested that the total amount of centriolar Asl $\left(A_{tot\;}\right)$should remain relatively unchanged between the *Plk4-NG* and *Plk4-NG*^*1/2*^ conditions (Data S1, second chart). Centriolar Asl levels were analyzed in embryos expressing Asl-mCherry in either WT versus *Plk4*^*1/2*^ conditions, and our findings showed that this was indeed the case (Figure S7A).

Next, the effects of reducing the genetic dose of *asl* by half (*asl*^*1/2*^ embryos—see Results and Discussion for details) were analyzed. Interestingly, the best-fit parameter values (*R*^*2*^ = 0.999) predicted that the total centriolar Asl levels (A_tot_) would be reduced by only ∼28% in *asl*^*1/2*^ embryos (Data S1, third chart). This value was therefore directly measured in embryos expressing either one or two copies of Asl-GFP (under the control of its own promoter in an *asl* mutant background). Encouragingly our findings showed that reducing the genetic dose of Asl-GFP by half led to a reduction of only ∼30% in centriolar Asl-GFP levels (Figure S7B). Moreover, the parameter values suggested that the concentration of Plk4 (incorporated in the *k*_1_ term) should not vary significantly between WT and *asl*^*1/2*^ conditions (Data S1, third chart). We confirmed this prediction using western blotting for Plk4-GFP and PeCoS for Plk4-NG (both transgenically expressed from their own promoters in a *Plk4* mutant background) in control and *asl*^*1/2*^ embryos (Figures S7C and S7D).

Taken together, these analyses indicate that our model can robustly describe the Plk4-NG oscillations under normal conditions (Figure 3C) and when the levels of either Plk4 or Asl are perturbed experimentally (Figures 4A and 4B). Moreover, the model makes several plausible predictions about the relative levels of these proteins in the perturbed conditions that are close to the levels that we measured experimentally.

Finally, the best-fit value of *k*_2_ (reflecting the kinase activity of individual Plk4 molecules) decreased slightly between cycles 11 to 12 and decreased more significantly between cycles 12 to 13 (by ∼9% and ∼37%, respectively); *k*_2_ also decreased when levels of Plk4 were genetically reduced in *Plk4-NG*^*1/2*^ embryos (by ∼25%)—but not when Asl levels were genetically reduced in *asl*^*1/2*^ embryos (Data S1; first, second and third charts). The molecular basis for this inferred decrease in kinase activity remains unknown, but we believe it is biologically plausible. We previously suggested that centriolar Plk4 was likely to integrate several inputs at the start of each cycle (from, for example, cell cycle regulators, or its activator Ana2/STIL) and adjust its kinase activity in response to the lengthening of S-phase during successive nuclear cycles (Aydogan et al., 2018). Moreover, our finding that Wee1 kinase, an important cell cycle regulator, can influence the Plk4 oscillation parameters in S-phase strongly supports this hypothesis (Figure 6).

#### Model 2: Generating robust Plk4 oscillations entrained by the CCO

The network described above was further extended to test the possibility of generating robust oscillations in centriolar Plk4 levels. To do so, we allow the Asl receptors to be dephosphorylated by a phosphatase at rate $k_{4}$ (Figure S4A). Subject to the constraint that there is no Plk4 bound to the Asl receptors initially (i.e., at the start of cycle 1), we model multiple cycles by allowing the phosphatase to be active only during mitosis, so that $k_{4}$ is nonvanishing only in this period. Therefore, the system reads:

(6) $$\frac{d\left[A_{0}^{*}\right]}{dt}=k_{1}\left[A_{0}\right]-k_{2}\left[A_{0}^{*}\right]$$

(7) $$\frac{d\left[A_{1}^{*}\right]}{dt}=k_{2}\left[A_{0}^{*}\right]-k_{2}\left[A_{1}^{*}\right]-k_{4}\left[A_{1}^{*}\right]$$

(8) $$\frac{d\left[A_{N}^{*}\right]}{dt}=k_{2}\left[A_{N-1}^{*}\right]-k_{3}\left[A_{N}^{*}\right]-k_{4}\left[A_{N}^{*}\right]$$

(9) $$\frac{d\left[A_{0}\right]}{dt}=k_{4}\left[A_{1}\right]-k_{1}\left[A_{0}\right]$$

(10) $$\frac{d\left[A_{1}\right]}{dt}=k_{4}\left[A_{2}\right]-k_{4}\left[A_{1}\right]$$

(11) $$\frac{d\left[A_{N}\right]}{dt}=k_{3}\left[A_{N}^{*}\right]-k_{4}\left[A_{N}\right]$$

subject to the initial conditions,

(12) $$\left[A_{0}\right]=1;\;\;\left[A_{1}\right]=\cdots =\left[A_{N}\right]=0;\;\;\left[A_{0}^{*}\right]=\cdots =\left[A_{N}^{*}\right]=0.$$

It is further assumed that the embryo is in mitosis for 30% of the total time in each nuclear cycle and all cycle times are kept constant. Hence, $k_{4}=0\;$ for 0<tmod T<0.7T and a positive constant for 0.7T<tmod T<T, where T is the period of the cell cycle. Values for the rate constants are determined by fitting the exact analytical solution in the S-phase of cycle 12 to the Lorentzian regression of the experimental data (*R*^*2*^ = 0.9870). In the first instance, we assumed that the cytosolic concentration of Plk4 remains constant over the nuclear cycles (see below). We plot the exact solution for the percentage of Asl-bound Plk4 molecules for a total of 14 nuclear cycles, as is the case in fly embryos. This minimal model was sufficient to generate sustained oscillations in centriolar Plk4 levels (Figure S4B; *k*_*4*_ = 0.0708).

As an alternative to the assumption that the cytosolic concentration of Plk4 is constant over the cycles, we also considered the case where the total number of Plk4 molecules in the embryo is kept constant (so any Plk4 degradation is balanced by new synthesis). In this model, as the number of centrioles, $N_{C}\left(t\right)$, increases at successive cycles so the number of available Plk4 molecules in the cytosol initially decreases during S-phase (as Plk4 binds to centriolar Asl receptors), and then increases (as Plk4 unbinds from Asl receptors). We estimate that there are $N_{P}$ = 10^5^ molecules of Plk4 in an embryo of 0.01mm^3^ volume (Markow et al., 2009); a concentration of ∼10nM, in agreement with that measured in human cells (Yamamoto and Kitagawa, 2019), but potentially higher than we infer from our observation that Plk4 levels are too low to be measured by FCS (as we cannot infer absolute protein concentration from our PeCoS experiments). To simulate the effect of centriole duplications, we double the number of centrioles each cycle and assume that, at each centriole duplication, the bound Plk4 (attached to Asl receptors) is equally split between the mother and separating daughter. To consider the Cdk/Cyclin trigger wave that sweeps through embryos (Deneke et al., 2016), it is assumed that the duplicated centrioles separate nearly synchronously over the last 10% of the time-window in each cycle. Based on our 3D-SIM microscopy data, we assume that each centriole has ∼30 Asl receptors, as we essentially only need to consider Plk4 binding to Asl at the site of centriole assembly (the model works well for between ∼20-80 receptors), and that there is a single centriole in cycle 1. With these modifications to the model, the system reads

(13) $$\frac{d\left[A_{0}^{*}\right]}{dt}=k_{1}\left[P\right]\left[A_{0}\right]-k_{2}\left[A_{0}^{*}\right]-\frac{1}{N_{C}}A_{0}^{*}\frac{dN_{C}}{dt}$$

(14) $$\frac{d\left[A_{1}^{*}\right]}{dt}=k_{2}\left[A_{0}^{*}\right]-k_{2}\left[A_{1}^{*}\right]-k_{4}\left[A_{1}^{*}\right]-\frac{1}{N_{C}}A_{1}^{*}\frac{dN_{C}}{dt}$$

(15) $$\frac{d\left[A_{N}^{*}\right]}{dt}=k_{2}\left[A_{N-1}^{*}\right]-k_{3}\left[A_{N}^{*}\right]-k_{4}\left[A_{N}^{*}\right]-\frac{1}{N_{C}}A_{N}^{*}\frac{dN_{C}}{dt}$$

(16) $$\frac{d\left[A_{0}\right]}{dt}=k_{4}\left[A_{1}\right]-k_{1}\left[A_{0}\right]+\;\frac{1}{N_{C}}A_{0}^{*}\frac{dN_{C}}{dt}$$

(17) $$\frac{d\left[A_{1}\right]}{dt}=k_{4}\left[A_{2}\right]-k_{4}\left[A_{1}\right]+\;\frac{1}{N_{C}}A_{1}^{*}\frac{dN_{C}}{dt}$$

(18) $$\frac{d\left[A_{N}\right]}{dt}=k_{3}\left[A_{N}^{*}\right]-k_{4}\left[A_{N}\right]+\;\frac{1}{N_{C}}A_{N}^{*}\frac{dN_{C}}{dt}$$

(19) $$\left[P\right]=1-\;\frac{N_{R}N_{C}\left(t\right)}{N_{P}}\sum_{n=0}^{N}A_{n}^{*}$$

subject to the initial conditions (12).

We plot the solution of the model for the percentage of Asl-bound Plk4 molecules, as well as the percentage of Plk4 molecules that remain in the cytoplasm, over 14 nuclear cycles (Figure S4F; *R*^*2*^ = 0.9871 and *k*_*4*_ = 0.0612).

We observe a small spike as the centrioles begin to separate at the end of each mitosis (Figure S4F). The spike is small, due to the slight asynchrony in centriole separations; if the centrioles *were* to separate all simultaneously, the concentration would instantly halve at this point, since the number of receptors would all double. Interestingly, these small spikes are consistent with our experimental observations (Figures 1A and S2A). Moreover, we emphasize that, for the first few nuclear cycles, almost all of the Plk4 remains in the cytoplasm since there are only a few centrioles. In the later cycles, however, the amount of Plk4 sequestered by the Asl receptors increases exponentially, as the number of centrioles increase by a factor of 2 in each cycle. Therefore, the rate at which the Asl receptors are able to recruit Plk4 from the cytoplasm decreases, resulting in a reduction in the amplitude of the Plk4 oscillation (Figure S4F). This feature of the model is also consistent with our experimental observations (Figures 1B and 1C).

#### Model 3: Stochastic duplications

Finally, we have also developed a discrete mathematical model, which is analogous to Model 2 described previously, in order to consider the possibility of stochastic centriole duplication in non-cycling embryos (as in those injected with dsRNA against cyclins A, B and B3; Figure S8G). As before, we assume that unphosphorylated Asl receptors bind Plk4 with high affinity until they become fully phosphorylated and Plk4 unbinds, and we initially assume that the Asl receptors can be dephosphorylated during mitosis. We model this system stochastically by defining the state vector for each receptor to be

(20) $$V=\left(A_{0},\;\cdots ,\;A_{N},\;A_{0}^{*},\cdots ,\;A_{N}^{*}\right)$$

At any given time, precisely one entry of V is equal to unity, corresponding to the state which the receptor is in at that moment, and all other entries are equal to zero. We allow the receptor to change state over time according to the transition matrix:

(21) $$M=\;\left[\begin{array}{cccccccccc} Q_{1} & 0 & 0 & \cdots & 0 & P_{1} & 0 & \cdots & \cdots & 0\\ P_{4} & Q_{4} & 0 & \ddots & 0 & 0 & 0 & \ddots & \ddots & \vdots \\ 0 & P_{4} & Q_{4} & \ddots & 0 & \vdots & \vdots & \ddots & \ddots & \vdots \\ \vdots & \ddots & \ddots & \ddots & \vdots & \vdots & \vdots & \ddots & \ddots & \vdots \\ 0 & \cdots & 0 & P_{4} & Q_{4} & 0 & 0 & \ddots & \ddots & \vdots \\ 0 & \cdots & \cdots & 0 & 0 & Q_{2} & P_{2} & 0 & \ddots & \vdots \\ \vdots & \ddots & \ddots & \vdots & \vdots & P_{4,2} & Q_{2,4} & P_{2,4} & 0 & \vdots \\ \vdots & \ddots & \ddots & \vdots & \vdots & 0 & \ddots & \ddots & \ddots & 0\\ \vdots & \ddots & \ddots & 0 & 0 & \vdots & \ddots & P_{4,2} & Q_{2,4} & P_{2,4}\\ 0 & \cdots & \cdots & 0 & P_{3,4} & 0 & \cdots & \;0 & P_{4,3} & Q_{3,4} \end{array}\right]$$

where

(22) $$P_{i}=1-e^{-k_{i}},\;\;\;\;\;\;\;P_{i,j}=\frac{k_{i}}{k_{i}+k_{j}}\left(1-e^{-\left(k_{i}+k_{j}\right)}\right),\;$$

(23) $$Q_{i}=e^{-k_{i}},\;\;\;\;\;\;\;Q_{i,j}=e^{-\left(k_{i}+k_{j}\right)},$$

describe the probabilities of a receptor changing state and remaining in the same state which arise in our model. We may allow the cytosolic Plk4 concentration to vary in this model by making the substitution $k_{1}\to \;k_{1}\left[P\right]$ and using (19) to compute [P] (evaluating the sum over all receptors being simulated). We also assume that, if a receptor is in a Plk4-bound state $\left(A_{n}^{*}\right)$ during the last 10% of a cycle, the Plk4 will unbind $\left(A_{n}^{*}\to \;A_{n}\right)$ with 50% probability during that time period in order to simulate mother-daughter separation.

In this model, each Asl receptor behaves as an independent oscillator—alternating between a Plk4-bound form that is being phosphorylated, and an unbound form that is being dephosphorylated. In the presence of the CCO, the individual Asl receptors generate coordinated oscillations because the CCO effectively synchronizes them every cycle by ensuring a coordinated burst of PPTase activity during mitosis. This activity is lost in the absence of the CCO, but instead we allow the PPTase to be active at a low, but constant, level (10% of the mitotic activity in cycling embryos). We plot the Asl-bound Plk4 levels for a total of 10 centrioles (each with 30 receptors as assumed above; Figure S8H). We observe that the centrioles are initially synchronized, since they all start in an unbound state, and display a single round of Plk4 binding. However, as time progresses, the Asl receptors lose synchrony, and each centriole exhibits stochastic, low-amplitude oscillations. Such oscillations may be sufficient to trigger duplications at individual centrioles, as evident from our experimental observations (Figures 5B–5F and S8B–S8F; Video S4).

All the equations used for mathematical modeling and regressions are available in the following web link: < https://github.com/RaffLab/centriole_oscillator_model >.

#### Fluorescence Correlation Spectroscopy (FCS)

##### FCS setup and measurements

Point FCS measurements were performed on a confocal Zeiss LSM 880 (Argon laser excitation at 488 nm and GaASP detector) with the Zen Black Software. A C-Apochromat 40x/1.2 W objective and a pinhole setting of 1AU were used. A laser power of 10 μW was used, and no photobleaching was observed during the measurements. The microscope was kept at 25°C using the Zeiss inbuilt heating insert P and the heating unit XL. A schematic overview of the methodology used is shown in Figure S5A, and a comparison of the average autocorrelation curves generated at the start of S-phase of nuclear cycles 11-14 is shown in Figure S5B.

The effective volume of the imaging setup was estimated to be 0.28 fL by averaging the estimate obtained by three independent methods, as described previously (Rüttinger et al., 2008): **1)** Measuring the concentration of a soluble Alexa Fluor 488 NHS Ester dilution series (100 nM, 10 nM, 1 nM and 0.1 nM); **2)** Measuring the diffusion time for Alexa Fluor 488 NHS Ester (same concentrations) in water at 25°C. The measured diffusion time was then compared to a previously reported diffusion coefficient for the Alexa Fluor 488 NHS Ester (Petrásek and Schwille, 2008); **3)** Imaging subresolution beads (FluoSpheres Carboxylate-Modified Microspheres, 0.1 μm) and determining the effective volume via Gaussian fitting with the *line tool* and *Z-axis profile* in ImageJ (Bethasda, USA).

Embryo collections (from mother flies expressing Asl-GFP under the control of its own promoter in an *asl* mutant background) were as described above, with the exception of using high precision 35 mm, high Glass Bottom μ-dishes (ibidi). Before every measurement, spherical aberrations were adjusted on the correction collar of the objective by maximizing the count-rate per molecule (CPM). At the beginning of S-phase in each cell cycle (when the old and new mother centrioles were separating), consecutive cytosolic measurements were made 6x for 10 s each at the centriolar plane of the embryo. Individual recordings where centrioles moved through the measurement spot, based on the highly erratic shape of the correlation curve (< 2% of all recordings), were discarded.

##### Autocorrelation analysis and post-acquisition curve fitting

The autocorrelation function, G(τ), was calculated during each measurement in the Zen Black software using the following equation:

$$G\left(\tau \right)=\frac{<\delta I\left(t\right)\cdot \delta I\left(t+\tau \right)>}{<\delta I{\left(t\right)}^{2}>}$$

where ⟨⟩ denotes a time average, δI(t) describes the intensity fluctuation at the time point *t*, and τ states the lag time of the autocorrelation.

All 10 s-recordings were then fitted with 8 different 3D diffusion models using the software FoCuS-Point (Waithe et al., 2016) with the following equation:

$$G_{3D}\left(\tau \right)=\sum_{k=1}^{D_{s}}A_{k}{\left(1+{\left(\frac{\tau }{\tau_{xyk}}\right)}^{\alpha^{k}}\right)}^{-1}{\left(1+\left(\frac{\tau }{AR^{2}\tau_{xyk}}\right)\right)}^{-1/2}$$

where *A*_*k*_ defines the fraction of a diffusing species for which the sum of all diffusing species equals 1, τ_*xy*_ describes the average residence time of the diffusing species in *V*_*eff*_, α accounts for anomalous subdiffusion within the cytoplasm, and *AR* is a structural parameter that describes the relationship among the x, y and z-axes of the excitation volume.

Dark states of the fluorophore were fitted with the following formula:

$$G_{T}\left(\tau \right)=1+\sum_{j=1}^{T_{s}}\frac{T_{j}}{1-T_{j}}\cdot e^{-\tau /\tau_{Tj}}$$

where *T* depicts the triplet population, and $\tau_{T}$ states the triplet correlation time during which the fluorophore stays in the dark state (Schönle et al., 2014).

The data was fitted within the boundaries of 4x10^−4^ ms and 1.5x10^3^ ms, and the dark states were restricted to 10-300 μs for the blinking state, and 1-10 μs for the triplet state. The models (**Ms**) were defined as the following: **M1)** 1 diffusing species (ds) 0 blinking states (bs) 0 triplet states (ts); **M2)** 1 ds 1bs 0ts; **M3)** 1 ds 0bs 1ts; **M4)** 1 ds 1bs 1ts; **M5)** 2 ds 0bs 0ts; **M6)** 2 ds 1bs 0ts; **M7)** 2 ds 0bs 1ts; **M8)** 2 ds 1bs 1ts. In all models, the structural parameter *AR* and the anomalous subdiffusion parameter α were kept constant at 5 and 0.7, respectively.

In order to avoid over-fitting the data, the most plausible model to describe the autocorrelation functions was selected using the Bayesian Information Criterion (BIC), which is based on the likelihood function, but introduces a penalty term for the complexity (number of variables) for the models (Schwarz, 1978). In this study, **M4** was the preferred model to describe Asl-GFP diffusion (Figure S5A_(iv)_). The concentration was calculated from the FoCuS-point fit data of the preferred model:

$$<N>=\frac{1}{G_{0}}\;,\;conc.=\frac{<N>}{V_{eff}}$$

where *N* states the average number of particles within the effective volume *V*_*eff*_, and *G*_*0*_ represents the height of the autocorrelation function at τ = 0.

#### FCS background corrections

In order to estimate the contribution of the background noise, 22 wild-type embryos were measured with the same laser intensity (10 μW) and in roughly the same plane and developmental stage as the Asl-GFP embryos. Despite no observable correlated background, the uncorrelated background contributed ∼30% of the total photon count rate, presumably due to the low concentration of cytosolic Asl-GFP and the high autofluorescence of the embryo itself (Figure S5_(v__)_). Background corrections were performed after the autocorrelation analysis by calculating the correction factor $\chi^{2}\;$using the following formula (Koppel, 1974):

$$\frac{1}{\chi^{2}}=\frac{1}{{\left(1+<b>/<f>\right)}^{2}}$$

$$<N>=\frac{1}{\chi^{2}G_{0}}$$

where $<b>$ denotes the average background and $<f>\;$states the average count rate of the sample.

#### Data restriction

In some FCS measurements a sudden drop in CPM was observed, possibly due to movements within the embryo or the embryo drifting away from the measurement plane. When this happened, a strong, often unreasonable increase in concentration was observed. These outliers were therefore discarded based on a ROUT outlier test (with the aggression factor Q = 1%), which was performed on all 10 s-long concentration measurements (the *red* data points in Figure S5_(vi)_). Only the embryos with at least 4x10 s recordings (after discarding outliers and erratically shaped ACFs) were included in the final analysis.

#### Peak Counting Spectroscopy (PeCoS)

FCS was not sensitive enough to investigate the cytosolic concentration of Plk4-NG, presumably because its concentration was too low. We therefore developed a new method that we term *Peak Counting Spectroscopy* (PeCoS) that allows the relative concentration of low abundance proteins to be measured accurately (Figure S6). PeCoS uses the same set up as the point FCS protocol described above, but it differs in terms of its data acquisition and analysis. In PeCoS, the intensity peaks, which are generated by a fluorophore moving through the effective volume, are counted as a proxy for concentration. Due to the low cytosolic concentration of the fluorescently-tagged protein of interest (e.g., Plk4-NG in this study), spherical aberrations could not be corrected within the same embryo where the measurements were taken. Therefore, embryos that express a bright fluorescent centriolar marker were positioned next to the experimental embryos on the same imaging dish and these were used for correction collar adjustment (Figure S6A_(i)_). Experimental recordings were then captured for 180 s (instead of 6x10 s), as the number of particles that pass through the field of view was usually very low. Before every measurement, the observation region was pre-bleached with the same laser intensity (10 μW) for 3 s to bleach away any potential immobile fraction.

Instead of autocorrelation analysis, the resulting intensity traces (Figure S6A_(iii)_) were quantified for their number of peaks, which originate from a fluorophore moving through the excitation volume and causing a detectable burst of photons. In order to determine the cut-off threshold, which was used to subtract the background noise, 40 control embryos were measured (Figure S6A_(iii)_). These control embryos were from mothers expressing Asl-mKate2 to allow measurements at the centriolar plane and at the right nuclear cycle stage (beginning of S-phase). “Mean + n^∗^SD” (where n = 1,2,3,…) of all control recordings was subtracted from each control recording, and the threshold that resulted in an average of less than five peaks (per 180 s control measurement) was subtracted from all intensity traces (Figure S6A_(iv-vi)_). This threshold was found to be a good compromise for minimizing the background noise without discarding too much information. A Python script was written in order to automate this procedure, which is available via < https://github.com/RaffLab/PeCoS >. The subtraction of “Mean + 8^∗^SD” resulted in an average peak count of 3.25 for the control recordings, and this was used for the background subtraction in all *in vivo* measurements. In the peak detection algorithm above, a peak was defined as any consecutive value (photon count) that surpasses the subtracted threshold (Figure S6A_(vi)_).

In order to assess the effective concentration range of the PeCoS methodology, two-fold dilution series of Alexa488 NHS Ester were measured, and for every sample, both the ACF and the number of peaks were calculated using FCS and PeCoS (where Background = Mean ± 23^∗^SD (water)), respectively. As expected, PeCoS did not perform well at high concentrations, where, presumably, too many particles move simultaneously through the excitation volume; at lower particle concentrations, however, (where FCS was no longer accurate) the number of peaks decreased in a nearly linear fashion (Figure S6B). To test the sensitivity of PeCoS under *in vivo* conditions, the cytosolic concentration of Plk4-NG was measured at the beginning of nuclear cycle 12 in embryos expressing either one (1x) or two (2x) copies of *Plk4-NG* in the *Plk4* mutant background. PeCoS analysis indicated a 90% increase in the number of peaks (per minute) in the 2x embryos compared to the 1x embryos, indicating the effectiveness of PeCoS in measuring the relative cytosolic concentration of low abundance proteins (Figure S6C).

### Quantification and Statistical Analysis

The details for quantification, statistical tests, sample numbers, definitions of center, and the measures for dispersion and precision are described in the main text, relevant figure legends, or relevant sections of STAR Methods. Significance in statistical tests was defined by p < 0.05. To determine whether the data values were normally distributed, a D’Agostino–Pearson omnibus normality test was applied. Prism 7 and 8 were used for all the modeling and statistical analyses.

## Supplemental Information

Table S1. Oligonucleotides Used in This Study, Related to Figures 1, 2, 3, 4, 5, and 6 and STAR Methods

Data S1. Parameter Values and Monte Carlo Analysis Used in the Mathematical Modeling, Related to Figures 3, 4A, and 4B and STAR Methods
