# Supplementary material for: An Autonomous Oscillation Times and Executes Centriole Biogenesis
Source: Cell. 2020 Jun 25;181(7):1566–1581.e27. doi: 10.1016/j.cell.2020.05.018 (PMC7327525; doi:10.1016/j.cell.2020.05.018)
Supplement: Data S1. Parameter Values and Monte Carlo Analysis Used in the Mathematical Modeling, Related to Figures 3, 4A, and 4B and STAR Methods [file mmc2.pdf]

Data S1. Parameter values and Monte Carlo analysis used in the mathematical modelling. (Related to Figures 3, 4A and 4B, and the STAR Methods)

The best-fit k_1, k_2, k_3, and $\hat{A}$ parameter values* for cycles 11-13 are listed below (related to Figure 3):

Parameters	Cycle 11	Cycle 12	Cycle 13
k_1	0.004762	0.003644	0.001272
k_2	0.03756	0.03443	0.02164
k_3	0.06906	0.06906	0.06906
$\hat{A}$	1012	940	1030
$\hat{A}_0$	701	637	557
A_{tot}	1713	1577	1587
R^2	0.998	0.9996	0.998

The best-fit k_1, k_2, k_3, and $\hat{A}$ parameter values* for the $Plk4^{1/2}$ experiment are listed below (related to Figure 4A):

Parameters	Control	$Plk4^{1/2}$
k_1	0.003862	0.001513
k_2	0.03699	0.02767
k_3	0.06906	0.06906
$\hat{A}$	4884	5607
$\hat{A}_0$	3152	2390
A_{tot}	8036	7997
R^2	0.9992	0.996

The best-fit k_1, k_2, k_3, and $\hat{A}$ parameter values* for the $asl^{1/2}$ experiment are listed below (related to Figure 4B):

Parameters	Control	$asl^{1/2}$
k_1	0.003437	0.002724
k_2	0.03246	0.03324
k_3	0.06906	0.06906
$\hat{A}$	1938	1408
$\hat{A}_0$	1298	950
A_{tot}	3236	2358
R^2	0.9996	0.9991

*For the definition of parameters, see *STAR Methods* under Mathematical modelling and Monte Carlo simulations.

Monte Carlo Analysis

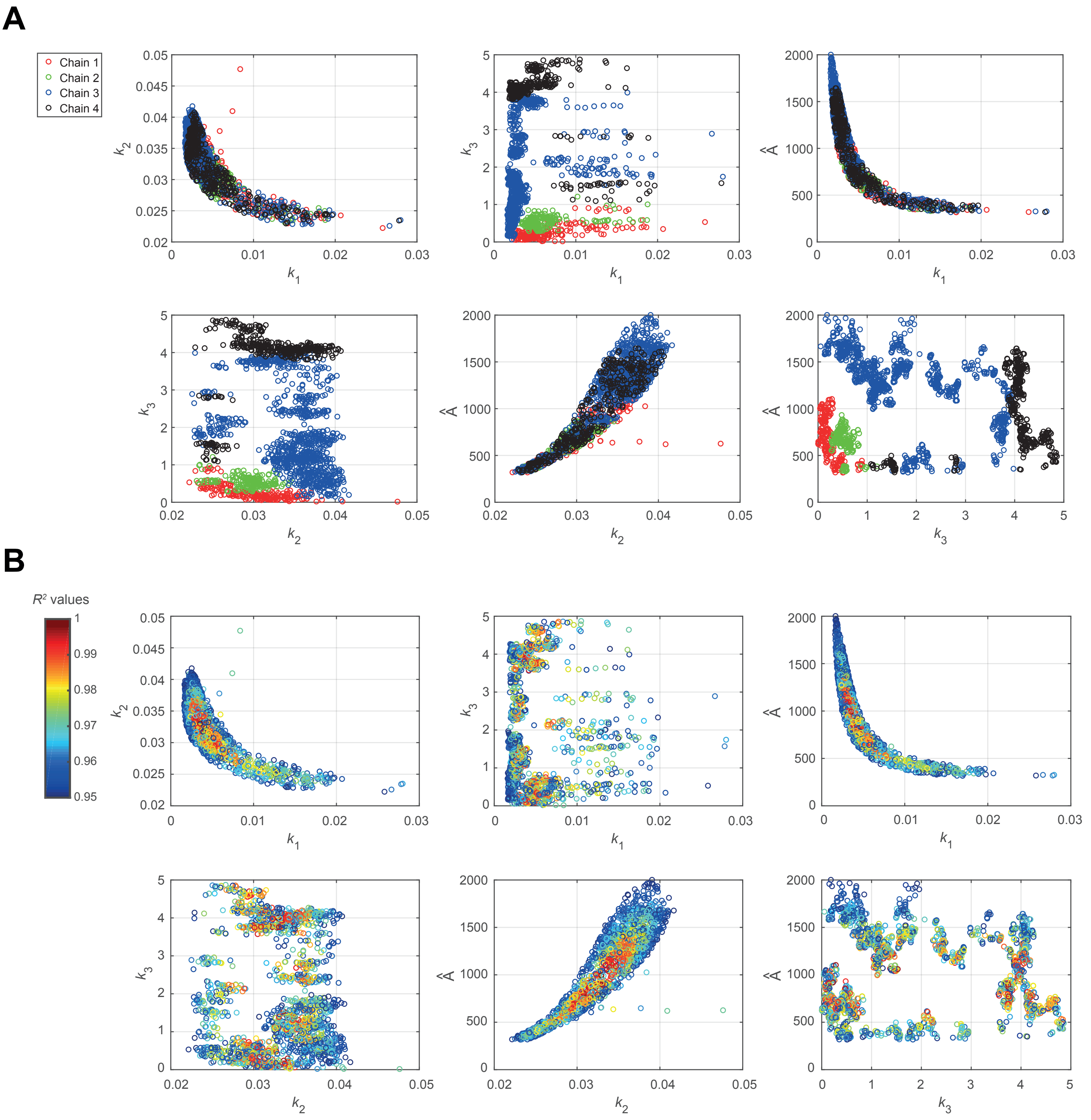

Monte Carlo analysis above (related to Figure 3 and the STAR Methods) characterises the parameter space of the mathematical model. Scatter plots in A show the results of four Markov chain Monte Carlo simulations (shown in different colours, as indicated). The six two-dimensional projections of the four-dimensional parameter space shown here show only those points that allowed the model to fit the data well (R^2>0.95). Scatter plots in B show the same data points as (A), but heat-mapped to show the R^2 value of each point. As in (A), only the data points with R^2>0.95 are shown. Points with a low R^2 value are shown in cool colours, while points with a high R^2 value are shown in warm colours (for the definition of parameters, see the mathematical modelling section in *STAR Methods*). These results indicate that most of the parameter values that allow the model to fit the data are likely to occupy a single, continuous, and relatively small region of the parameter space. The only exception is k_3; the likely reasons for this are also discussed in *STAR Methods*.

Starting parameter values and proposal acceptance rates for each of four Markov chains (used in the Metropolis-Hastings Markov chain Monte Carlo analysis above) are listed below:

Parameters	Chain 1	Chain 2	Chain 3	Chain 4
k_1	0.0035	0.01	0.1	0.0035
k_2	0.035	0.09	0.2	0.035
k_3	0.05	0.5	0.1	4
$\hat{A}$	1000	500	1500	1500
**Acceptance**	36%	36%	29%	37%
